# [^11^C]mHED PET follows a two-tissue compartment model in mouse myocardium with norepinephrine transporter (NET)-dependent uptake, while [^18^F]LMI1195 uptake is NET-independent

**DOI:** 10.1186/s13550-020-00700-7

**Published:** 2020-09-29

**Authors:** Linjing Mu, Stefanie D. Krämer, Geoffrey I. Warnock, Achi Haider, Susan Bengs, Giovanni Cartolano, Dominic S. Bräm, Claudia Keller, Roger Schibli, Simon M. Ametamey, Philipp A. Kaufmann, Catherine Gebhard

**Affiliations:** 1 Department of Nuclear Medicine, University Hospital Zurich, Raemistrasse 100, 8091 Zurich, Switzerland; 2 Department of Chemistry and Applied Biosciences, Radiopharmaceutical Sciences, Institute of Pharmaceutical Sciences, ETH Zurich, 8093 Zurich, Switzerland; 3 Center for Molecular Cardiology, University of Zurich, 8952 Schlieren, Switzerland

**Keywords:** [^11^C]meta-hydroxyephedrine ([^11^C]mHED), [^18^F]LMI1195 ([^18^F]flubrobenguane), Norepinephrine transporter (NET, SLC6A2), Cardiac sympathetic innervation imaging, Small animal PET, Kinetic modelling

## Abstract

**Purpose:**

Clinical positron emission tomography (PET) imaging of the presynaptic norepinephrine transporter (NET) function provides valuable diagnostic information on sympathetic outflow and neuronal status. As data on the NET-targeting PET tracers [^11^C]meta-hydroxyephedrine ([^11^C]mHED) and [^18^F]LMI1195 ([^18^F]flubrobenguane) in murine experimental models are scarce or lacking, we performed a detailed characterization of their myocardial uptake pattern and investigated [^11^C]mHED uptake by kinetic modelling.

**Methods:**

[^11^C]mHED and [^18^F]LMI1195 accumulation in the heart was studied by PET/CT in FVB/N mice. To test for specific uptake by NET, desipramine, a selective NET inhibitor, was administered by intraperitoneal injection. [^11^C]mHED kinetic modelling with input function from an arteriovenous shunt was performed in three mice.

**Results:**

Both tracers accumulated in the mouse myocardium; however, only [^11^C]mHED uptake was significantly reduced by excess amount of desipramine. Myocardial [^11^C]mHED uptake was half-saturated at 88.3 nmol/kg of combined mHED and metaraminol residual. After [^11^C]mHED injection, a radiometabolite was detected in plasma and urine, but not in the myocardium. [^11^C]mHED kinetics followed serial two-tissue compartment models with desipramine-sensitive *K*_1_.

**Conclusion:**

PET with [^11^C]mHED but not [^18^F]LMI1195 provides information on NET function in the mouse heart. [^11^C]mHED PET is dose-independent in the mouse myocardium at < 10 nmol/kg of combined mHED and metaraminol. [^11^C]mHED kinetics followed serial two-tissue compartment models with *K*_1_ representing NET transport. Myocardial [^11^C]mHED uptake obtained from PET images may be used to assess cardiac sympathetic integrity in mouse models of cardiovascular disease.

## Introduction

Cardiac sympathetic activation is an essential physiological stress response adapting cardiac performance to increased workload [1]. However, persistent sympathetic firing is also a key neurohormonal abnormality with major prognostic implications in many cardiovascular conditions [2, 3]. Indeed, the contribution of sympathetic hyperactivity to disease progression and adverse outcomes in heart failure patients is well established [2]. Moreover, patients with acute coronary syndrome show an elevated sympathetic activity, potentially owing to the interaction of the sympathetic nervous system with inflammatory processes [4]. Nevertheless, despite an improved understanding of autonomous dysbalance in ischemic heart disease and congestive heart failure, significant knowledge gaps remain with respect to the detrimental effects of cardiac innervation and denervation on cardiovascular endpoints [5, 6].

The development of radioligands targeting the presynaptic norepinephrine transporter (NET, "uptake-1", SLC6A2), such as the benzylguanidine analogue [^123^I]*meta*-iodobenzylguanidine ([^123^I]mIBG) for single-photon emission computed tomography (SPECT) and the norfenefrine analogue [^11^C]meta-hydroxyephedrine ([^11^C]mHED, [^11^C]HED) for positron emission tomography (PET) has enabled the non-invasive assessment of the neurohumoral axis [7]. However, in clinical imaging, [^123^I]mIBG is limited by the moderate resolution of SPECT. The use of [^11^C]mHED is challenged by the short half-life of ^11^C (20 min), spill-over from the liver in preclinical imaging, and the fact that [^11^C]mHED uptake in the mouse heart depends on the co-injected dose of its precursor metaraminol [8]. Thus, the PET tracer [^18^F]LMI1195 ([^18^F]flubrobenguane) has recently been introduced [9]. This F-18 offers a longer physical half-life (110 min) than C-11 as well as improved spatial resolution in tissue due to lower energy of the β^+^ decay [10]. Despite the successful clinical validation of both [^11^C]mHED and [^18^F]LMI1195, detailed studies of these tracers in mice are scarce. This represents a major knowledge gap given that mouse models of cardiac disease provide a unique opportunity to identify molecular mechanisms accounting for the adverse effects of sympathetic dysregulation in cardiovascular risk and disease conditions.

We aimed to portray the uptake mechanisms of [^11^C]mHED and [^18^F]LMI1195 in the mouse myocardium. Myocardial uptake of [^11^C]mHED was analysed in detail, by taking into account tracer molar activity, biotransformation, and uptake kinetics. Finally, the utility of standardized uptake value (SUV) as a measure of NET function in the mouse myocardium was addressed.

## Materials and methods

All chemicals, unless otherwise stated, were purchased from Sigma-Aldrich (Buchs, Switzerland), Acros Organics (Reinach, Switzerland), or Merck (Darmstadt, Germany) and used without further purification. Metaraminol bitartrate, the precursor for C-11 radiolabelling, was obtained from ABX advanced biochemical compounds GmbH (Radeberg, Germany). For NET inhibition, desipramine HCl (Sigma-Aldrich, Buchs, Switzerland) was dissolved in water for injection at 10 mg/ml (corresponding to 9 mg/ml as base). The reference compound **9** (LMI1195, Additional file 1) and the tosylate activated precursor **7** (Additional file 1) used for F-18 radiolabelling were synthesized following the modified approach described by Purohit et al. [11]. The radiosyntheses of [^11^C]mHED and [^18^F]LMI1195 are described in Additional file 1.

### Animals

Ten-week-old female FVB/N mice were obtained from Janvier Labs (Le Genest-Saint-Isle, France). The animals had free access to food and water. The mice were scanned at 12–14 weeks of age (20–24 g body weight).

### Ex vivo radiometabolite studies

Mice were injected awake with formulated [^11^C]mHED (26–173 MBq) via the tail vein. Animals were anaesthetized with 5% isoflurane in oxygen/air (1:1) and euthanized by decapitation at the indicated time points to collect blood, urine, and heart tissue. Plasma was separated from blood by centrifugation (5000×*g* for 5 min at 4 °C). Plasma and urine were each mixed with equal volumes of ice-cold acetonitrile (MeCN) for protein precipitation. The dissected heart was homogenized in 2 ml PBS, and proteins were precipitated with 2 ml ice-cold MeCN. The samples were centrifuged (5000×*g*, 4 °C), and the supernatants were filtered and analysed by radio-UPLC (Waters Acquity UPLC HSS T3 1.8 µm) with the following separation conditions: 10 mM NH_4_HCO_3_ (solvent A), MeCN (solvent B); 0.0–1.0 min, 5–30% B; 1.0–1.7 min, 30% B; 1.7–1.8 min, 30–5% B and 1.8–3.0 min, 5% B at a flow rate of 0.5 ml/min (retention time: 1.75 min). The fraction of parent tracer radioactivity to total radioactivity in plasma (*f*_parent_) was calculated as the ratio of radioactivity in the [^11^C]mHED peak and total detected radioactivity in the chromatogram. Equation 1 was fit to the data, using the nls function of *R* (R-project, version 3.6.1).

1 $${f}_{\mathrm{parent}}={q}_{1}\times \mathrm{exp}\left({-\,q}_{2}\times \frac{{t}^{2}}{{t}^{2}+{q}_{3}^{2}}\times t\right)+(1-{q}_{1})\times \mathrm{exp}({-\,q}_{4}\times t)$$

In Eq. 1, *q*_1_ to *q*_4_ are the fit parameters (*q*_3_ was estimated and fixed) and *t* is the time. Equation 1 was empirically defined, taking into account the exponential functions of time under linear kinetics and a lag phase for metabolite formation.

### Ratio of plasma to whole blood radioactivity

For determining activity concentration ratios of plasma to whole blood in mice, 11–90 MBq [^11^C]mHED were injected intravenously (i.v.) via the tail vein in awake mice. After anaesthesia, animals were euthanized at the indicated time points and blood was collected. An aliquot was centrifuged as described above to separate plasma from blood cells. Plasma and whole blood activity concentrations (Bq/ml) were determined by a gamma counter (Wizard 1480; Perkin Elmer) to calculate the plasma/whole blood activity concentration ratio (*ρ*_plasma/blood_). Bi-exponential function (Eq. 2) was fit to *ρ*_plasma/blood_ over time using the nls function of *R*.

2 $$\rho_{\mathrm{plasma}/\mathrm{blood} }={r}_{1}\times \mathrm{exp}\left({-\,r}_{3}\times t\right)-{r}_{2}\times \mathrm{exp}\left({-\,r}_{4}\times t\right)+{r}_{2}$$

In Eq. 2, *r*_1_ to *r*_4_ are the fit parameters and *t* is the time.

### PET/CT acquisition

PET/computed tomography (CT) scans were performed with a calibrated SuperArgus PET/CT scanner (Sedecal, Madrid, Spain) with an axial field of view of 4.8 cm and a spatial resolution of 1.6–1.7 mm (full width at half maximum) [12]. Animals were under anaesthesia with ~ 2.5% isoflurane in oxygen/air (1:1) for tracer injection into the tail vein and during the scan. For scans with NET inhibition, 20 mg desipramine HCl was injected intraperitoneally (i.p.) 10 min before tracer i.v. injection when the animal was already under anaesthesia. For scans without kinetic modelling, tracer was injected into mouse tail vein on scanner bed and the scan was started 1 min p.i. For kinetic modelling, [^11^C]mHED was injected using the shunt system (see “Input function” section) on the scanner bed. Depth of anaesthesia was monitored by measuring the respiratory rate (SA Instruments, Inc., Stony Brook, USA). Body temperature was monitored by a rectal probe and kept at 37 °C by a heated air stream (37 °C). Table 1 shows further details, including group sizes, injected dose, and scan durations. PET scans were followed by a CT for anatomical orientation.

**Table 1:** Details of the PET/CT scans

	[^11^C]mHED baseline	[^11^C]mHED desipramine	[^11^C]mHED kinetic modelling	[^18^F]LMI1195 baseline	[^18^F]LMI1195 desipramine
Group size	5	6	3	6	6
Body weight (g)	20.2–23.8	19.6–22.9	27.5–28.9	19.1–24.5	20.5–22.8
Activity (MBq)	2.8–11.5	3.5–13.9	9.2–11.0	3.3–10.8	1.2–8.6
Dose (nmol/kg)^a^	9.2.0–61.6	9.6–50.0	1.5–2.0	1.6–18.3	3.0–11.8
Scan duration (min)	60	60	60	90	90
Scan start p.i. (min)	1	1	− 1	1	1

^a^Dose in nmol/kg includes the dose of mHED and residual metaraminol

Two [^11^C]mHED scans (one baseline, one for kinetic modelling) were excluded from further analysis due to technical problems before or during the scan, respectively (not included in Table 1). PET data were reconstructed using 2-D ordered subsets expectation maximization in user-defined time frames with a voxel size of 0.3875 × 0.3875 × 0.775 mm^3^ (*x*, *y*, axial). Images, volumes-of-interest, and the respective time-activity curves (TAC) as well as NIfTI format files for further analysis with *R* were generated with the dedicated software PMOD (version 3.9, PMOD Technologies Ltd., Zurich, Switzerland). The nominal voxel size of the NIfTI format files was 0.243 mm in all dimensions. Tissue radioactivity was either expressed as kBq/cm^3^ or normalized to the injected dose per g body weight (kBq/g) and expressed as SUV, assuming a tissue density of 1 g/cm^3^. All activities were decay-corrected to the time of tracer injection.

### Input function

For kinetic modelling, an arteriovenous shunt system was applied to record the coincidences of arterial blood (Twilite, Swisstrace, Zurich, Switzerland) simultaneously with the PET data acquisition (Additional file 1) [13]. The resulting blood coincidences were transformed to Bq/ml with a calibration factor determined from simultaneous measurements of an [^11^C]mHED solution in the PET scanner and the blood counter. Next, the blood Bq/ml was multiplied with fitted Eq. 2 to calculate the plasma activity concentration. Finally, the plasma Bq/ml was multiplied with fitted Eq. 1 to receive the parent tracer Bq/ml in plasma, which is the input function [14].

### Definition of volume-of-interest for quantitative analyses

**Fig. 1:**
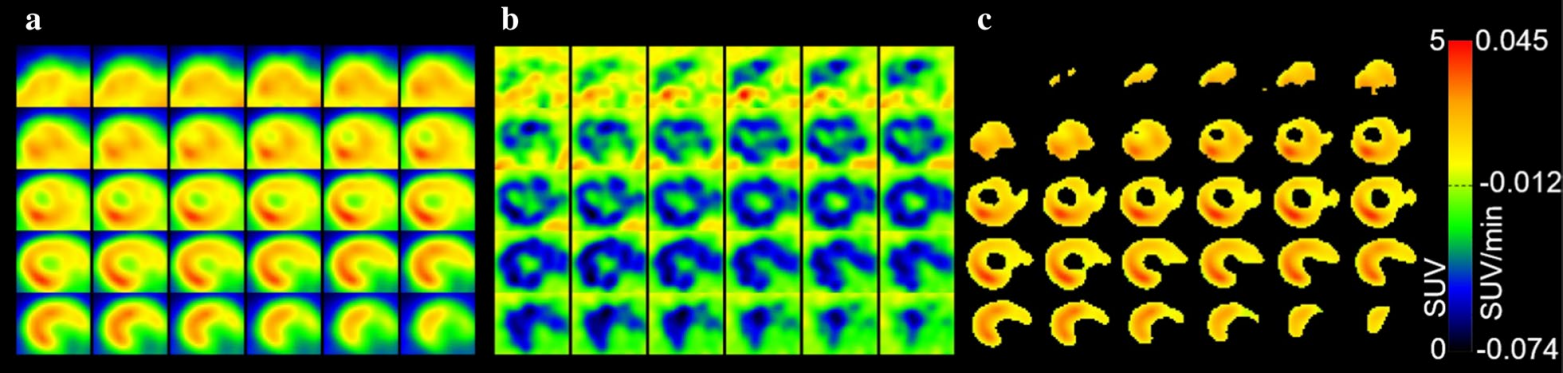
Strategy for the definition of the volume-of-interest for myocardial [^11^C]mHED uptake. **a** Transversal planes of a cropped volume including the heart, from posterior (top left) to anterior (bottom right). SUV_1–61 min_ according to colour scale. **b** Voxel-wise slopes SUV/time between 11 and 48 min p.i. Slopes according to colour scale. Voxels with slopes ≥ − 0.012 SUV/min were excluded from the volume-of-interest, assuming spill-over from liver (liver radioactivity was increasing over time). **c** From the remaining voxels, the volume with highest SUV between 3 and 11 min p.i., corresponding to 0.5% of the individual body weight (i.e. body weight (g) × 0.005 cm^3^/g). Scan with 9.2 MBq [^11^C]mHED (1.5 nmol/kg, including metaraminol mass), body weight 27.6 g

For quantitative analyses, PET data in NIfTI file format were read with the *R* package oro.nifti version 0.9.1, and a cube of 36 × 30 × 30 voxels (8.7 × 7.3 × 7.3 mm^3^) including the heart as judged from the PET and CT data was cropped. A liver mask was defined including all voxels with a slope ≥ − 0.012 SUV/min between 11 and 48 min p.i. (the slope limit was empirically defined by visual inspection of the results, Fig. 1). PET data corresponding to the liver mask were excluded. The myocardium mask was defined from the remaining data as the volume with highest SUV between 3 and 11 min p.i., corresponding to 0.5% of the individual body weight [15]. A typical myocardium data set is shown in Fig. 1. Average SUV was calculated for the indicated time windows (stated as SUV_start time–end time_). The SUV of the neck region was obtained from a spherical region of interest with 3 mm radius, placed over the shoulders, close to the spine.

### Dose dependency of myocardial [^11^C]mHED uptake

The dose of combined administered [^11^C]mHED and metaraminol (precursor for radiosynthesis) at half-maximal NET saturation in nmol/kg (*D*_50_) was calculated from the myocardial SUV_1–31 min_ at various doses (*D*). SUV_1–31 min_ of scans with 20 mg/kg desipramine HCl (66,000 nmol/kg, full NET saturation) and of scans for kinetic modelling was included (Table 1; total *n* = 14). Fit parameters were SUV_1–31 min_ at indefinitely low dose (SUV_max_), SUV_1–31 min_ at full NET saturation, i.e. SUV_1–31 min_ for nondisplaceable tracer (SUV_min_) and *D*_50_, according to Eq. 3 which was fit to the data with the function nls of *R*.

3 $${\mathrm{SUV}}_{1{-}31\mathrm{min}}=\left({\mathrm{SUV}}_{\mathrm{max}}-{\mathrm{SUV}}_{\mathrm{min}}\right)\times \frac{{D}_{50}}{{D}_{50}+D}+{\mathrm{SUV}}_{\mathrm{min}}$$

### Compartment modelling and statistical analysis

PET kinetic modelling with *R* was performed with a one- and three two-tissue compartment models according to Eq. 4,

4 $${C}_{\mathrm{PET}}=\left(1-{v}_{\mathrm{b}}\right)\times {W}_{\mathrm{model}}\otimes {C}_{\mathrm{a}}+{v}_{\mathrm{b}}\times {C}_{\mathrm{b}}$$

where *C*_PET_ is the activity concentration in the PET image, either as kBq/cm^3^ or normalised as SUV, *v*_b_ is the partial volume of blood in the region-of-interest (potentially including interstitial space), *W*_model_ is the model-dependent weighting function. *C*_a_ is the parent tracer activity concentration in the arterial plasma (input function, kBq/ml, or SUV), and *C*_b_ is the whole blood activity concentration as calculated from the blood coincidences, in kBq/cm^3^ or as SUV.

**Fig. 2:**
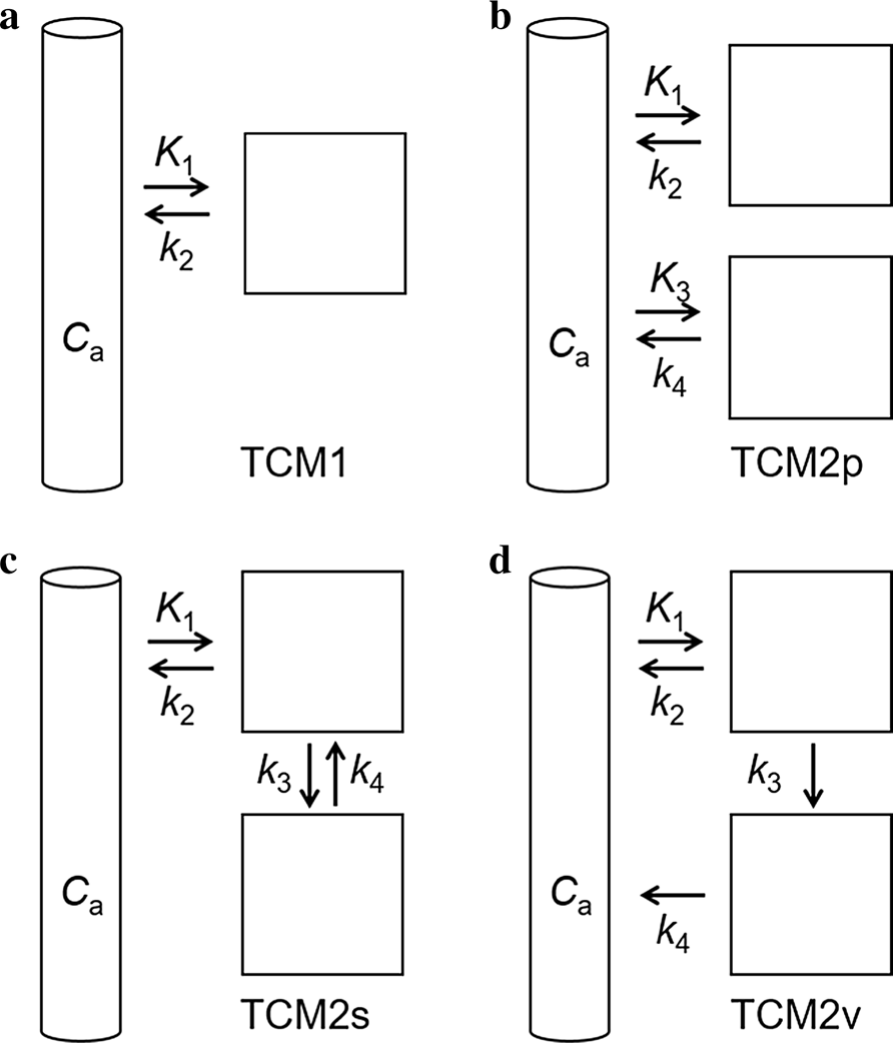
Compartment models evaluated in this study. **a** One-tissue compartment model (TCM1). **b** Model with two parallel tissue compartments (TCM2p) both directly accessible from the input compartment. **c** Two-tissue compartment model with the two-tissue compartments in series (TCM2s), as applied for neuroreceptor PET. *K*_1_ or *k*_3_ would represent NET uptake. In the first case, *k*_3_ would represent uptake into storage vesicles by vMAT2. In the latter case, *K*_1_ would represent extravasation into interstitium. **d** Similar as **c** but with the second-tissue compartment releasing to the input compartment (*k*_4_) rather than to the first tissue compartment. In this model, *K*_1_ would represent NET uptake, *k*_3_ uptake into storage vesicles by vMAT2, and *k*_4_ release from storage vesicles into interstitial space (TCM2v, v for release from vesicles). *C*_a_, [^11^C]mHED activity concentration in arterial plasma (input function)

The evaluated tissue compartment models are shown in Fig. 2. *W*_TCM1_ for the one-tissue compartment model (TCM1, Fig. 2a) corresponds to *K*_1_ × exp(− *k*_2_ × *t*), with *t* as time, *K*_1_ and *k*_2_ the clearance term from plasma to tissue and rate constant back, respectively. *W*_TCM2p_ and W_TCM2s_ for the two-tissue compartment models with parallel (TCM2p, Fig. 2b) and serial (TCM2s, Fig. 2c) tissue compartments, respectively, were according to Eqs. 5–10 [16]. In the case of the TCM2p, *K*_1_, *k*_2_, *K*_3_, and *k*_4_ correspond to *φ*_1_, *θ*_1_, *φ*_2_, and *θ*_2_, respectively, of Eq. 5. *W*_TCM2v_ for the two-tissue compartment model in Fig. 2d (TCM2v) was numerically solved from the differential equation system in Eqs. 11 and 12 with the *R* package deSolve and the ode function, with 1-s time intervals.

5 $${W}_{\mathrm{TCM}2\mathrm{p}/\mathrm{s}}={\varphi }_{1}\times \mathrm{exp}\left(-\,{\theta }_{1}\times t\right)+{\varphi }_{2}\times \mathrm{exp}\left(-\,{\theta }_{2}\times t\right)$$

6 $$\delta =\sqrt{{\left({k}_{2}+{k}_{3}+{k}_{4}\right)}^{2}-4\times {k}_{2}\times {k}_{4}}$$

7 $${\theta }_{1}=\left({k}_{2}+{k}_{3}+{k}_{4}+\delta \right)/2$$

8 $${\theta }_{2}=\left({k}_{2}+{k}_{3}+{k}_{4}-\delta \right)/2$$

9 $${\varphi }_{1}={K}_{1}\times \left({\theta }_{1}-{k}_{3}-{k}_{4}\right)/\delta$$

10 $${\varphi }_{2}={K}_{1}\times \left({\theta }_{2}-{k}_{3}-{k}_{4}\right)/-\delta$$

11 $$\frac{\mathrm{d}{C}_{1}}{\mathrm{d}t}=-\left({k}_{2}+{k}_{3}\right)\times {C}_{1}, \quad {\mathrm{with}}\quad {C}_{1}(0)={K}_{1}$$

12 $$\frac{\mathrm{d}{C}_{2}}{\mathrm{d}t}=+{k}_{3}\times {C}_{1}-{k}_{4}\times {C}_{2}$$

Besides the transfer clearance or rate constants *K*_1_, *k*_2_, *k*_3_ (or *K*_3_ for TCM2p), and *k*_4_, *v*_b_ was included as a parameter to fit. Equation 3 was fit to the experimental TACs applying the optim minimization function of *R*.

Fits were run with multiple starts with a matrix of 101 sets of random start parameters between lower and upper limits for *K*_1_, *k*_2_, *k*_3_ (or *K*_3_)_,_ *k*_4_, and *v*_b_ (Additional file 1: Table S1). The activity concentrations in the individual tissue compartments were simulated by numerically solving the respective differential equation systems for TCM2s and TCM2v and from the analytical solutions for TCM2p (the two exponential terms of Eq. 5). Calculations were performed in parallel on a 32 core workstation. Best fits were chosen based on the sum of squared residues (SSR) between predicted and experimental TACs. Best fits were present more than once in the 101 runs, suggesting that the global minimum was found for each scan and model. Correlations between parameters were calculated with the lm, cor, and cor.test functions of *R* and were considered significant at *p* < 0.05.

## Results

### Chemistry and radiochemistry

The organic synthesis and radiolabelling of the PET tracers [^11^C]mHED and [^18^F]LMI1195 are described in Additional file 1. [^11^C]mHED was synthesized by direct N-methylation of metaraminol with [^11^C]methyl triflate in 5% water in acetonitrile at room temperature [17]. Molar activities were in the range of 48–153 GBq/µmol (including residual metaraminol) at the end of synthesis (*n* = 22). The radiochemical purity was greater than 99%, as confirmed by HPLC analysis. The identity of the tracer was confirmed by co-injection with nonradioactive mHED. The total synthesis time from end of bombardment (EOB) was 35–40 min.

The radiosynthesis of [^18^F]LMI1195 was carried out by a one-pot, two-step reaction sequence consisting of nucleophilic fluorination of the corresponding tosylate precursor and followed by cleavage of the protecting groups [9]. The molar activities were in the range of 45–95 GBq/μmol at the end of synthesis. [^18^F]LMI1195 was obtained in excellent radiochemical purity, and the average synthesis time was approximately 80 min from the EOB.

### [^11^C]mHED and [^18^F]LMI1195 PET and specificity for NET

**Fig. 3:**
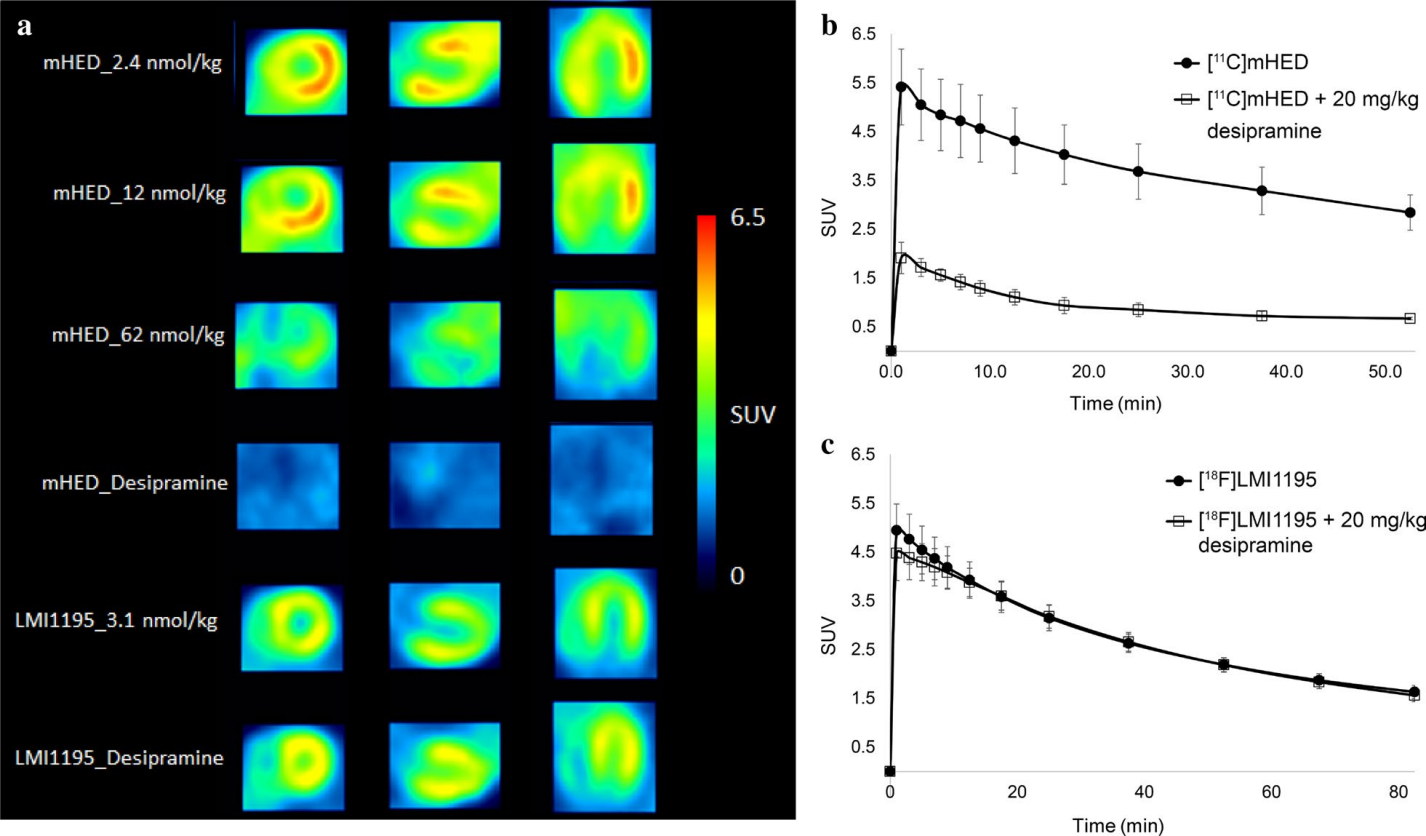
Standard PET images of myocardium after i.v. injection of [^11^C]mHED or [^18^F]LMI1195 in FVB/N mice. **a** Representative short-axis (left column), horizontal long-axis (middle column), and vertical long-axis (right column) PET images at various extents of NET saturation with metaraminol (mHED with indication of total mass of [^11^C]mHED and metaraminol) or desipramine (20 mg/kg desipramine HCl as indicated) and [^18^F]LMI1195 under baseline conditions or with desipramine. SUV was averaged from 1 to 31 min p.i. **b** TACs after injection of [^11^C]mHED alone (9.2–16.6 nmol/kg, including metaraminol mass; *n* = 3) and [^11^C]mHED (9.6–10.5 nmol/kg) + 20 mg/kg desipramine HCl (*n* = 3). **c** TACs after injection of [^18^F]LMI1195 alone (mass range, see Table 1; *n* = 6) and [^18^F]LMI1195 (mass range, see Table 1) + 20 mg/kg desipramine HCl (*n* = 6). Mean values with SD

Mouse myocardium was clearly visualized after [^11^C]mHED or [^18^F]LMI1195 administration (Fig. 3a). For assessing their uptake mechanism, excess amount of desipramine, a NET inhibitor with strong affinity (*K*_i_ value of 2.76 nM) [18] was used as the blocker. As shown in Fig. 3b and Fig. 3a, c blocking effect was observed for [^11^C]mHED but not for [^18^F]LMI1195. These results indicate distinct myocardial uptake mechanisms for [^11^C]mHED and [^18^F]LMI1195 in mice, with high selectivity for NET in the case of [^11^C]mHED but not [^18^F]LMI1195. Given that 80–90% of norepinephrine (NE) in the synaptic cleft is taken up by NET in the human heart [19, 20], we subsequently focused on characterizing the kinetics of the NET-selective tracer, namely [^11^C]mHED.

### Dose dependency of [^11^C]mHED PET in mice

We performed a dose dependency study to define the upper limit of injected mass for linear kinetics, i.e. proportionality between PET signal and activity dose. Since metaraminol, the precursor for radiosynthesis, and mHED have similar binding affinities for NET (*D*_50_ in the range of 90–100 nmol/kg in the rat left ventricle wall) [21], we did not distinguish between mHED and metaraminol for calculating the total injected dose in nmol/kg.

**Fig. 4:**
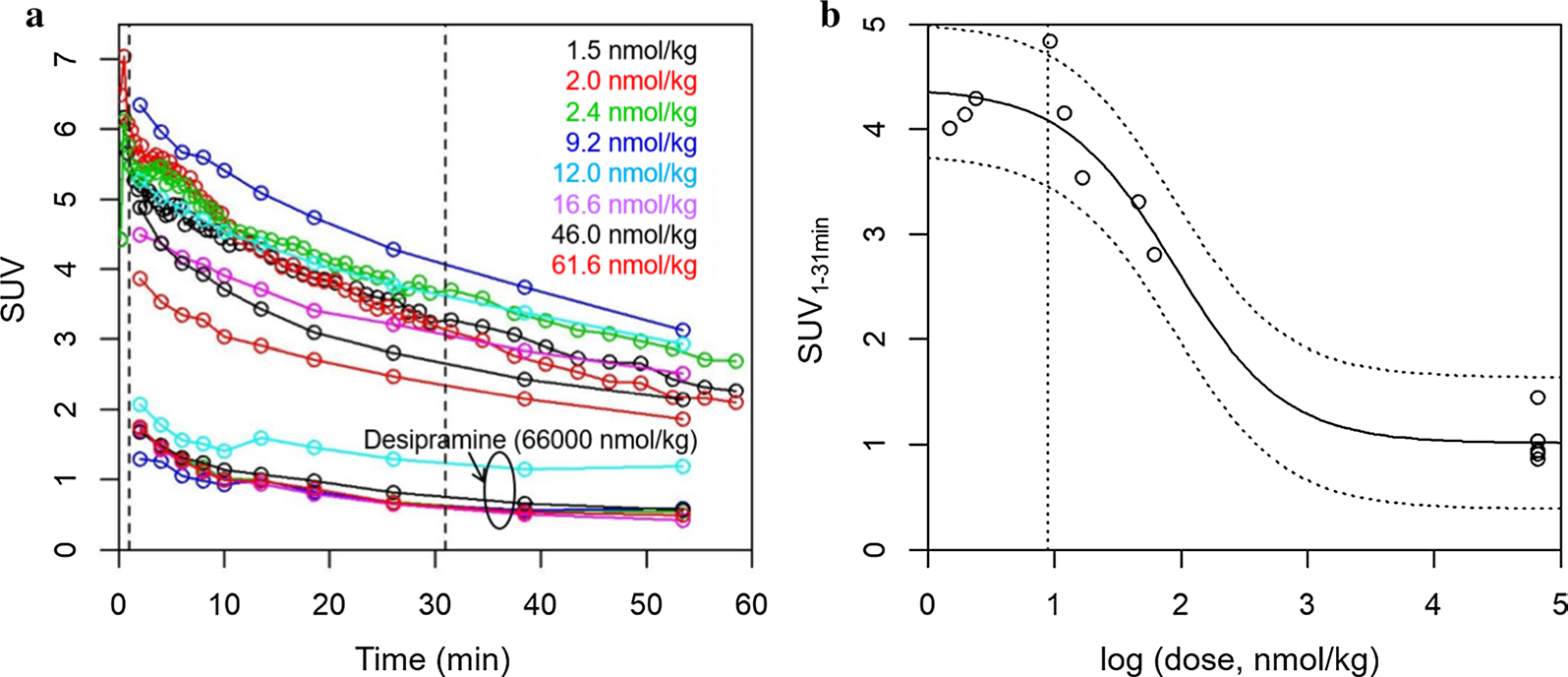
Dose dependency of [^11^C]mHED uptake in the mouse myocardium. **a** TACs (SUV) of myocardium at the indicated dose of combined [^11^C]mHED and metaraminol. Desipramine, 66′000 nmol/kg, corresponds to 20 mg/kg desipramine HCl. Broken vertical lines indicate 1 and 31 min, respectively, the time window for SUV_1–31 min_ averaging. TACs with shorter time intervals are from the scans for kinetic modelling. **b** SUV_1–31 min_ was plotted against the dose on logarithmic scale (base 10). Solid line, fit according to Eq. 3 with fit *D*_50_ = 88.3 nmol/kg. Broken lines, 95% confidence intervals. Broken vertical line indicates 1/10 *D*_50_

As shown in Figs. 3a and 4, myocardial [^11^C]mHED SUV decreased with increasing dose of injected mass. The relationship between SUV_1–31 min_ and the injected total nmol/kg is shown in Fig. 4b. Scans with desipramine blocking were included to define the lower plateau of the saturation function (SUV_min_ in Eq. 3), corresponding to unspecific signal at full saturation of NET. Nonlinear regression analysis according to Eq. 3 revealed a *D*_50_ of 88.3 nmol/kg (SE 30.3). Fit SUV_max_ and SUV_min_ were 4.4 and 1.0, respectively, at a volume-of-interest size of 0.5% body volume. Including data from the complete scan duration (SUV_1–61 min_) revealed *D*_50_ = 89.1 nmol/kg (SE 32.2). We chose ~ 1/10 of *D*_50_ as the maximal tolerated dose for further studies, i.e. 10 nmol/kg. At this dose, SUV is reduced in theory by 10% as compared to indefinitely low dose. This is within the experimental error (Fig. 4). The initial average concentration in the region-of-interest at 10 nmol/kg would be ~ 50 nM (product of SUV and dose in nmol/kg). The SUV_1–31 min_ of the neck region was independent of the dose and similar to myocardium SUV_min_ (Additional file 1: Fig. S1).

### Radiometabolite and plasma-to-whole blood radioactivity ratio

**Fig. 5:**
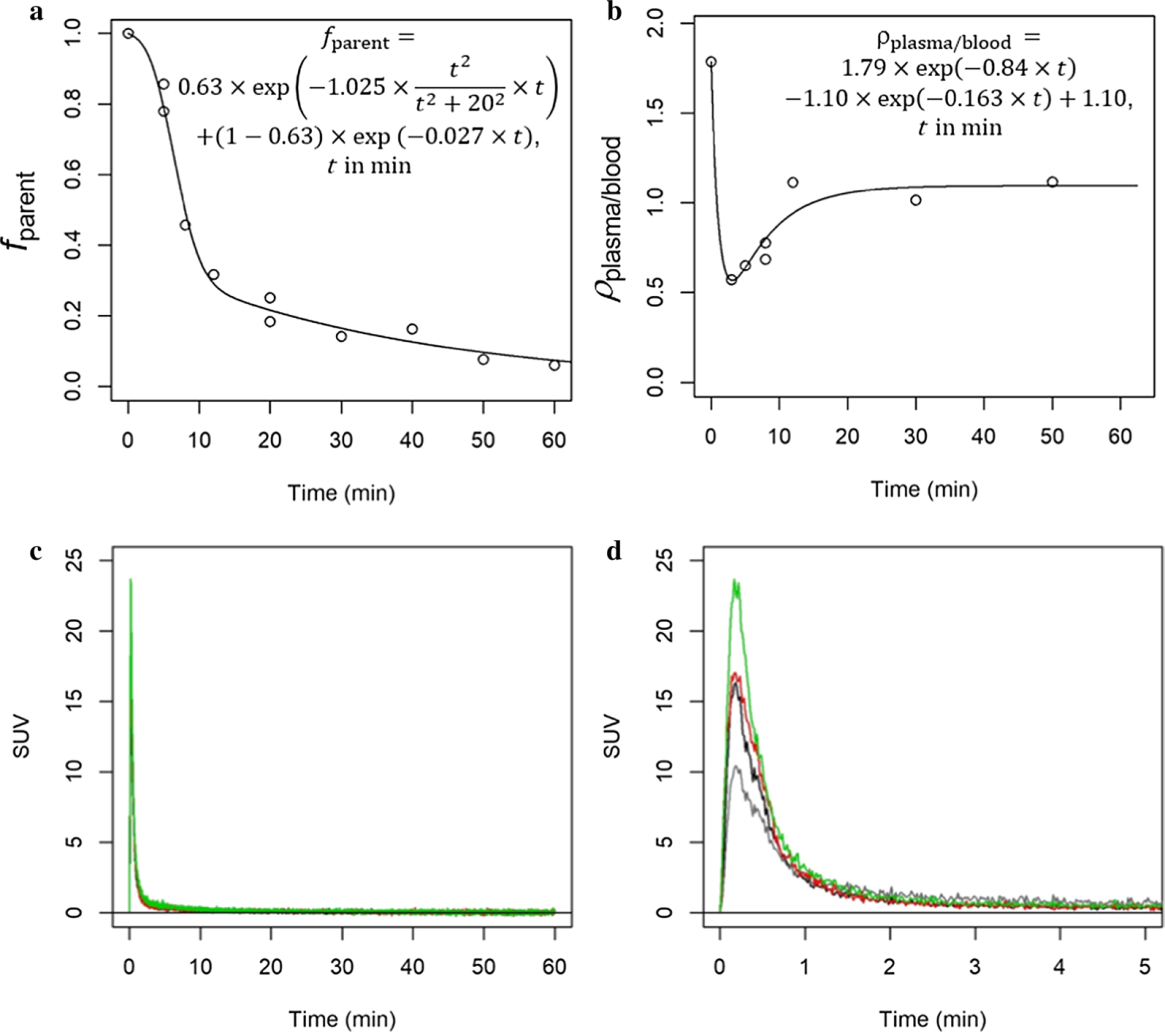
Generation of the input function. **a** Radioactivity fraction of parent tracer in plasma over time. **b** Ratio of activity concentration between plasma and whole blood over time. Lines are fit functions according to Eqs. 1 and 2, respectively, and as shown in the graphs. **c** Generated normalized (SUV) input functions of the 3 scans with blood coincidence measurements. **d** as **c**, emphasizing the first 5 min. Black, red, green, input functions; grey (lowest peak), whole blood SUV corresponding to the black input function (second lowest peak)

After [^11^C]mHED i.v. injection, we detected a radiometabolite in plasma and urine which was more polar than the parent tracer. Figure 5a shows *f*_parent_ in plasma over time. In the myocardium, only parent [^11^C]mHED and no radiometabolite were detected (Additional file 1: Fig. S2). The ratio *ρ*_plasma/blood_ is shown in Fig. 5b. At time *t* = 0, *ρ*_plasma/blood_ was assumed to be 1/(1-Hkt), with Hkt, hematocrit of 0.458 [15], resulting in an initial *ρ*_plasma/blood_ of 1.85. The ratio decreased to ~ 0.6 at 3 min and then increased to a constant value of ~ 1 from ~ 20 min onwards. These data indicate substantial blood cell binding or uptake of [^11^C]mHED, while the radiometabolite distributed equally between blood cells and plasma. The [^11^C]mHED accumulation in blood cells may result from ATP-driven uptake of [^11^C]mHED by the vesicular monoamine transporter 2 (vMAT2) into platelets [22]. Figure 5c and Fig. 5d show the resulting input functions of the three scans for kinetic modelling after correcting the blood activity concentration with *ρ*_plasma/blood_, *f*_parent_, and normalization to injected dose in kBq/g to reveal SUV.

### Myocardial [^11^C]mHED follows a two-tissue compartment model

**Fig. 6:**
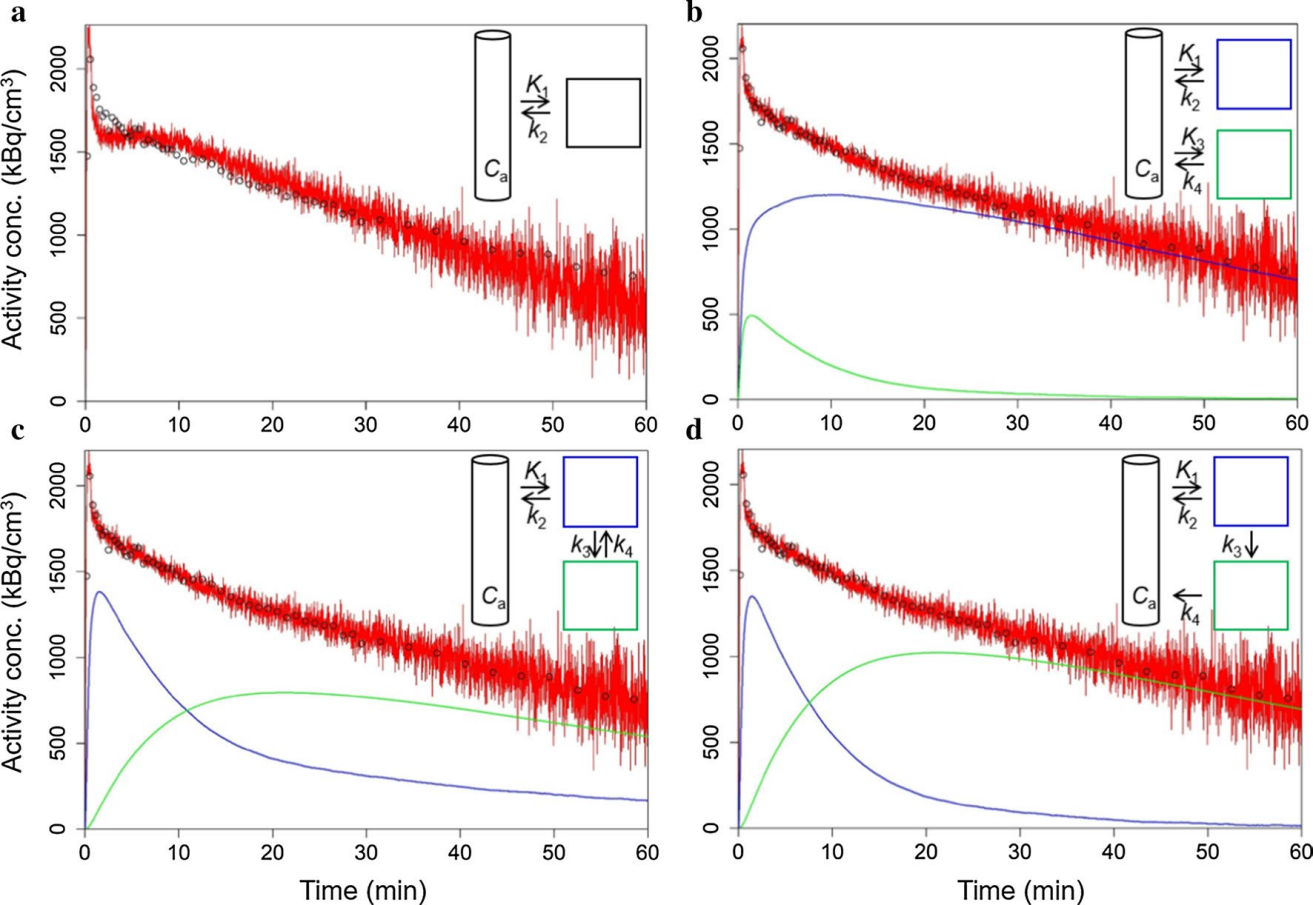
Representative TAC (black symbols) and fits (red lines) to a one-tissue compartment model (TCM1, **a**) and three two-tissue compartment models, **b** TCM2p, **c** TCM2s, **d** TCM2v. Tracer activity concentrations were simulated for the individual compartments as indicated in the insets (green and blue lines). The volume-of-interest corresponded to 0.5% of the individual body volume

We compared the results of kinetic modelling applying a one- and three two-tissue compartment models according to Fig. 2. Results were satisfactory by visual inspection for the two-tissue compartment models (TCM2) but not for the one-tissue compartment model (TCM1, Fig. 6a). Figure 6b (TCM2p) assumes two parallel mechanisms of tracer uptake. This would take into account potential desipramine-insensitive uptake as seen for [^18^F]LMI1195 besides desipramine-sensitive presynaptic uptake by NET. Figure 6c shows the two-tissue compartment model with the tissue compartments in series (TCM2s). In the model described in Fig. 6d (TCM2v), *k*_3_ describes tracer uptake into storage vesicles by vMAT2 and *k*_4_ represents tracer release from synaptic vesicles to the interstitial space without kinetic distinction between tracer in plasma and interstitial space, i.e. tracer exchange between plasma and interstitial space is faster than the temporal resolution of the scanner. As expected for the three two-tissue compartment models, best fits generated equal SSR and both *K*_1_ and *k*_2_ were equal for TCM2s and TCM2v. Table 2 shows the averaged fit parameters of the 3 scans.

**Table 2:** Fit parameters of four-compartment models for [^11^C]mHED myocardial uptake in mice (mean of 3 independent scans, SD in parentheses)

	TCM1	TCM2p	TCM2s	TCM2v
*K*_1_ (ml/min/cm^3^)	0.868 (0.113)	0.499 (0.153)	0.947 (0.146)	0.947 (0.146)
*k*_2_ (1/min)	0.029 (0.01)	0.0125 (0.0034)	0.0672 (0.0062)	0.0672 (0.0062)
*k*_3_ (1/min),	–	–	0.0541 (0.0263)	0.0669 (0.0344)
*K*_3_ (ml/min/cm^3^)	–	0.448 (0.114)	–	–
*k*_4_ (1/min)	–	0.134 (0.040)	0.0254 (0.0117)	0.0125 (0.0034)
*v*_b_ (–)	0.497 (0.006)	0.464 (0.035)	0.464 (0.035)	0.464 (0.035)

### NET-specific [^11^C]mHED uptake is represented by ***K***_***1***_

**Fig. 7:**
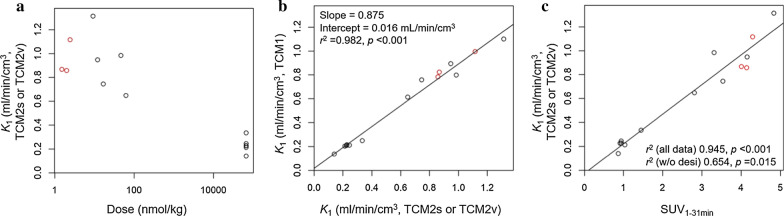
**a** Dose dependency and desipramine sensitivity of *K*_1_ in the TCM2s and TCM2v. Dose of combined [^11^C]mHED and metaraminol up to 61.6 nmol/kg. The highest dose in the plot (66 µmol/kg) corresponds to 20 mg desipramine HCl to fully saturate NET. **b** *K*_1_ of TCM1 correlated with *K*_1_ of the more complex TCM2s or TCM2v. Slope, intercept, *r*^2^, and *p* as indicated. **c** *K*_1_ of the TCM2s and TCM2v correlated with SUV_1–31 min_. SUV_1–31 min_ may serve as a surrogate parameter for NET transport activity. *r*^2^ and *p* for the data including scans with desipramine ("all data", regression lines) and without desipramine scans ("w/o desi"), as indicated. Note that for the estimation of *K*_1_, 11 of the 14 scans were analysed with a surrogate input function. Red symbols, scan with an input function

To identify the desipramine-sensitive process and the most adequate model, we modelled the TACs of all scans shown in Fig. 4, based on one of the three available input functions (red curve in Fig. 5d). We assumed that mHED, metaraminol, and desipramine had no major influence on the input function. This assumption is supported by the dose- and desipramine-independent SUV_1–31 min_ of the neck region (Additional file 1: Fig. S1). The parameter *v*_b_ was limited between 0.4 and 0.5, according to the fit *v*_*b*_ of the three scans with input function (Table 2). Two representative simulations are shown in Additional file 1: Fig. S3. We compared the fit parameters with the injected total mass ([^11^C]mHED, metaraminol, and desipramine), between the models and with the model-independent SUV_1–31 min_, respectively. The correlations are shown in Fig. 7 and Additional file 1: Figs. S4–S6.

For all applied models, *K*_1_ was significantly reduced by NET saturation with desipramine (66 µmol/kg total mass in Fig. 7a), indicating that NET transport is the rate-determining step for the process described by *K*_1_. Tracer exchange between plasma and interstitial fluid was thus faster than NET transport. The TCM2p model was evaluated to identify a potential desipramine-insensitive uptake process. In this model, however, both *K*_1_ and *K*_3_ were significantly reduced by desipramine, indicating that this model is not adequate.

After excluding TCM1 and TCM2p, TCM2s and TCM2v remained as candidate models. These two models revealed the same value for *K*_1_ (0.947 ± 0.146 ml/min/cm^3^, Table 2) and are thus both valid for the quantification of NET function. *K*_1_ may even be estimated with a one-tissue compartment model. *K*_1_ averaged from the 3 scans with an input function was 0.87 ± 0.11 ml/min/cm^3^ for TCM1 (Table 2), < 10% lower than determined with TCM2s/TCM2v. Over all 14 analysed scans, *K*_1_ of TCM1 correlated with *K*_1_ of TCM2s/TCM2v with a slope of 0.875 and *p* < 0.001 (Fig. 7b).

### SUV as a parameter to assess NET function

Myocardial SUV was averaged from 1 to 31 min (SUV_1–31 min_) or 1 to 61 min (SUV_1–61 min_). Additional file 1: Fig. S7 shows their high correlation with *r*^2^ = 0.997 and *p* < 0.001. The comparisons between the fit parameters and averaged SUV in Fig. 7c and Additional file 1: Fig. S6 show that SUV_1–31 min_ as well as SUV_1–61 min_ correlated with *K*_1_ in all models. In the TCM2s and TCM2v, no other parameter correlated significantly with SUV_1–31 min_ or SUV_1–61 min_. It should be taken into account that 11 of the 14 scans were fit with a surrogate input function and that parameters were poorly defined for scans with desipramine due to the low PET signal. Irrespective of these limitations, the data suggest that [^11^C]mHED SUV is a valuable parameter to assess NET function.

## Discussion

While both [^11^C]mHED and [^18^F]LMI1195 have been used for (pre)clinical imaging of cardiac sympathetic innervation, species differences have been reported for [^18^F]LMI1195. [^18^F]LMI1195 cardiac uptake was desipramine-sensitive in rabbits, nonhuman primates, and human subjects, but not in rats [9, 23]. In rats, myocardial uptake was assigned to the extra-neuronal "uptake-2" mechanism. The suspected transporter protein(s) mediating "uptake-2" is/are also present in the mouse myocardium [7, 24]. We now extend these findings by demonstrating that [^18^F]LMI1195 is accumulating in the mouse myocardium, but its uptake is not related to NET function, in agreement with the findings in rats. In contrast, the efficient blocking of [^11^C]mHED uptake by desipramine in our study indicates that [^11^C]mHED is highly NET-specific in the mouse myocardium, and "uptake-2" of [^11^C]mHED is absent or negligible. Reported *D*_50_ was 91 nmol/kg and 132 nmol/kg in the rat and mouse myocardium, respectively, which is consistent with the *D*_50_ determined in our study. Our findings clearly identify [^11^C]mHED as a suitable tracer for in vivo imaging of NET function in the mouse heart. Of note, however, [^11^C]mHED myocardial uptake was dependent on the mass dose of combined mHED and the synthesis precursor metaraminol, as previously reported [8, 21]. Thus, it is advisable to limit the injected mass dose for quantitative imaging to 10 nmol/kg, which includes both product and residual metaraminol.

After [^11^C]mHED injection, we detected one polar radiometabolite in the mouse plasma, while more than two polar radiometabolites were previously found in plasma of rats and guinea pigs [17, 21]. In line with these studies, we detected only intact [^11^C]mHED in the mouse myocardium. The determined plasma to whole blood activity ratios after [^11^C]mHED injection is comparable with the reported values for rats [21].

Both models TCM2s and TCM2v were suitable for determining NET transport kinetics (*K*_1_). TCM2v may represent more adequately the physiological situation of vesicle uptake by vMAT2 (*k*_3_) and release from the vesicles into the interstitial space (*k*_4_) [25]. Vesicular uptake of [^11^C]mHED was demonstrated in rabbits by Nomura et al. [26], as the vMAT2 inhibitor reserpine reduced [^11^C]mHED myocardial radioactivity at later time points during the scan. Based on our data, we cannot distinguish whether [^11^C]mHED is released to intra- or extracellular space or both. As the TCM2s model has an analytical solution for the weighting function allowing shorter computing time, and is a commonly used model for PET, it may perfectly serve as a model to quantify mouse myocardial [^11^C]mHED uptake by NET. Even a one-tissue compartment model may be applied to estimate *K*_1_, as shown by the good correlation between the respective *K*_1_ and *K*_1_ from the TCM2s and TCM2v models.

The fact that *K*_1_ was the desipramine-dependent parameter in our study indicates that the exchange of tracer between plasma and extracellular space was too fast to be resolved from the PET data. *K*_1_ thus describes the kinetics of [^11^C]mHED uptake from plasma to nerve terminal and not from extracellular space to nerve terminal. In this combined process, NET uptake is the rate-limiting step as concluded from the strong dose dependency. Tracer in the extracellular compartment may be included in our term *v*_b_ × *C*_b_ in Eq. 4. The maximal possible clearance parameter from plasma to extracellular space equals the plasma flow [27]. Myocardial blood flow under 2% isoflurane anaesthesia was 16.9 ml/min/g in a high-resolution spin labelling magnetic resonance imaging study [28], corresponding to ~ 9 ml/min/cm^3^ plasma flow, about ninefold higher than *K*_1_ in our study. As the rate-determining process in *K*_1_ was NET transport rather than plasma flow, minor to moderate changes in myocardial blood flow should not affect *K*_1_ [27]. Nevertheless, *K*_1_ may be affected by drastically reduced myocardial perfusion, the closer the plasma flow approaches *K*_1_.

In humans, [^11^C]mHED uptake is stronger affected by myocardial blood flow than in mice (under isoflurane anaesthesia), as the mean human resting myocardial blood flow is ~ 1 ml/min/g [29] (0.5–0.6 ml/min/g plasma flow), while *K*_1_ of [^11^C]mHED uptake was up to 0.6 ml/min/g in a recent PET study [30]. Patients with ongoing ischemia may display regionally reduced [^11^C]mHED uptake due to significant perfusion defects [31]. In clinical [^11^C]mHED PET, information on myocardial blood flow is therefore required for quantifying NET function and is usually obtained from myocardial perfusion imaging prior to [^11^C]mHED PET [5].

The recording of the blood radioactivity, e.g. image-derived from the left ventricle lumen, is recommended for a robust readout in clinical [^11^C]mHED PET. This allows calculating the retention index corresponding to the ratio of the regional tissue activity concentration of [^11^C]mHED (kBq/ml tissue) averaged from 30 to 40 min and the integral of the whole-blood TAC from 0 to 40 min (kBq·min/ml blood), both derived from the PET images [32]. In mice, recording of an arterial input function is technically challenging. For studies without blood counts recording, we suggest SUV as a surrogate of NET uptake activity. Consistent with our data, SUV has previously been advocated as a reproducible parameter for [^11^C]mHED NET uptake in the rat myocardium [33].

There are limitations to this study that should be pointed out. First, only three animals were scanned with a protocol of kinetic modelling that included an arteriovenous shunt system which may compromise the conclusions drawn from our study. Second, 11 of the 14 scans were fit with a surrogate input function and parameters were poorly defined for scans with desipramine due to the low PET signal. Third, the animals utilized in this study were healthy mice of identical genetic background and size. In disease models or genetically modified animals, the influence of body weight and other confounders may affect systemic tracer distribution and, thus, hamper the application of SUV. In the latter case, it might be more accurate to apply the percentage of injected dose per cm^3^ (%ID per cm^3^) instead of SUV for comparison of NET function. Irrespective of these limitations, the close relation of *K*_1_ with SUV seen in our study suggests that myocardial [^11^C]mHED uptake in mice is a reliable parameter to assess NET function. In contrast, myocardial [^18^F]LMI1195 uptake was not related to NET function in mice. Further studies are required to better define the mechanism by which [^18^F]LMI1195 accumulates in the mouse myocardium.

## Conclusion

Our data indicate that [^11^C]mHED kinetics follow a two-tissue compartment model in the mouse myocardium, with *K*_1_ describing NET transport kinetics. Our results further unveil a close relation of *K*_1_ with SUV in normal mice, suggesting that SUV might be a reliable parameter for NET function, thereby eliminating the need for arterial blood sampling. Given that NET function is essential for cardiac sympathetic integrity, our findings encourage a broader application of [^11^C]mHED PET imaging in murine models in cardiovascular research.

## Supplementary information

**Additional file 1: Supplementary Material and Methods, Tables, Figures.** LMI1195 precursor and reference compounds synthesis. Synthesis of [^18^F]LMl1195. Synthesis of [^11^C]mHED. Arteriovenous shunt and blood coincidence counting. Supplementary Table S1. Lower and upper limits of fit parameters. Supplementary Figure S1. SUV1-31min of the neck region. Supplementary Figure S2. Radio-UPLC profiles of heart and urine extracts. Supplementary Figure S3. TACs and fits under partial or full NET saturation. Supplementary Figure S4. Dose-dependence of fit parameters. Supplementary Figure S5. Comparisons of fit clearance and mass transfer rate constants. Supplementary Figure S6. Comparison of fit parameters with SUV. Supplementary Figure S7. Correlation between SUV_1-31min_ and SUV_1-61min_

## Data Availability

The datasets used and/or analysed during the current study are available from the corresponding author on reasonable request. R scripts are available on request from the authors.
