# Supplementary material for: [11C]mHED PET follows a two-tissue compartment model in mouse myocardium with norepinephrine transporter (NET)-dependent uptake, while [18F]LMI1195 uptake is NET-independent
Source: EJNMMI Res. 2020 Sep 29;10:114. doi: 10.1186/s13550-020-00700-7 (PMC7524946; doi:10.1186/s13550-020-00700-7)
Supplement: Supplementary file 1 — Additional file 1: Supplementary Material and Methods, Tables, Figures. LMI1195 precursor and reference compounds synthesis. Synthesis of [18F]LMl1195. Synthesis of [11C]mHED. Arteriovenous shunt and blood coincidence counting. Supplementary Table S1. Lower and upper limits of fit parameters. Supplementary Figure S1. SUV1-31min of the neck region. Supplementary Figure S2. Radio-UPLC profiles of heart and urine extracts. Supplementary Figure S3. TACs and fits under partial or full NET saturation. Supplementary Figure S4. Dose-dependence of fit parameters. Supplementary Figure S5. Comparisons of fit clearance and mass transfer rate constants. Supplementary Figure S6. Comparison of fit parameters with SUV. Supplementary Figure S7. Correlation between SUV1-31min and SUV1-61min [file 13550_2020_700_MOESM1_ESM.pdf]

[^{11}C]mHED PET follows a two-tissue compartment model in mouse myocardium with norepinephrine-transporter (NET) dependent uptake while [^{18}F]LMI1195 uptake is NET-independent

Linjing Mu[1,2*], Stefanie D. Krämer[2*], Geoffrey I. Warnock[1,3], Achi Haider[1,3], Susan Bengs[1,3], Giovanni Cartolano[2], Dominic S. Bräm[2], Claudia Keller[2], Roger Schibli[2], Simon M. Ametamey[2], Philipp A. Kaufmann[1], Catherine Gebhard[1,3]

[1]Department of Nuclear Medicine, University Hospital Zurich, 8091 Zurich, Switzerland
[2]Radiopharmaceutical Sciences, Institute of Pharmaceutical Sciences, Department of Chemistry and Applied Biosciences, ETH Zurich, Vladimir-Prelog Weg 4, 8093 Zurich, Switzerland
[3]Center for Molecular Cardiology, University of Zurich, 8952 Schlieren, Switzerland

*****: L. Mu and S.D. Krämer contributed equally to this manuscript
Corresponding author: Catherine Gebhard; Email: Catherine.Gebhard@usz.ch

SUPPORTING INFORMATION

MATERIALS AND METHODS

All chemicals, unless otherwise stated, were purchased from Sigma-Aldrich (Buchs, Switzerland), Acros Organics (Reinach, Switzerland) or Merck (Darmstadt, Germany), and used without further purification. Nuclear magnetic resonance spectra were recorded on a Bruker 400 MHz and a Bruker 500 MHz spectrometer with the corresponding deuterated solvent signals as internal standards. Chemical shifts are reported in parts per million (ppm) relative to tetramethylsilane (0.00 ppm). Values of the coupling constant (J) are given in hertz (Hz); the following abbreviations are used in this section for the description of the ^{1}H-NMR: singlet (s), doublet (d), triplet (t), quartet (q), multiplet (m), doublet of doublets (dd), and bs (broad signal). The chemical shifts of complex multiplets are given as the range of their occurrence. High-resolution mass spectra (HR-MS) were recorded with a Bruker FTMS 4.7 T BioAPEXII (ESI).

For radiolabelling product quality control, radio-HPLC chromatography was performed using an Agilent 1100 system with Gina software, equipped with UV multi-wavelength and Raytest Gabi Star detectors. Semi-preparative HPLC system was used for product purification. It is a Merck-Hitachi L6200A system equipped with Knauer variable wavelength detector and Eberline radiation detector. For the *ex vivo* stability studies, an ultra-performance liquid

chromatography (UPLC™) system from Waters and an attached Berthold co-incidence detector (FlowStar LB513) were used.

LMI1195 precursor and reference compounds synthesis

Synthesis of 3-bromo-4-((tert-butyldimethylsilyl)oxy)benzaldehyde

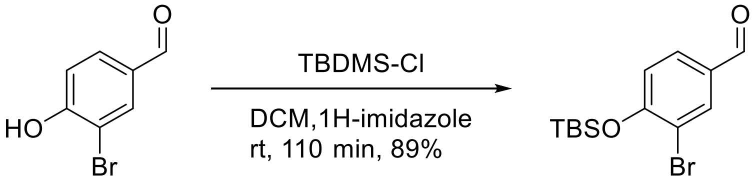

1 **2**

3-Bromo-4-hydroxybenzaldehyde **1** (1.5 g, 7.47 mmol) was dissolved in dry dichlomethane (DCM, 60 ml) and 1H-imidazole (1.35 g, 19.86 mmol) and TBDMS-Cl (1.50 g, 9.92 mmol) were added. The reaction mixture was stirred at room temperature for 110 min and extracted with DCM (4×25 ml). The organic fractions were combined, dried over $MgSO_4$ and concentrated under vacuum to yield a yellowish oil. The crude product was purified by column chromatography (3 → 5% EtOAc in hexane) to afford 3-bromo-4-(tert-butyl-dimethylsilanyloxy)-hydroxybenzaldehyde **2** (2.1 g, 89%) as a colorless oil.

^{1}H-NMR (400 MHz, CDCl3) δ 9.84 (s, 1H), 8.07 (d, J = 2.0 Hz, 1H), 7.72 (dd, J = 8.4, 2.0 Hz, 1H), 6.97 (d, J = 8.4 Hz, 1H), 1.05 (s, 9H), 0.30 (s, 6H).

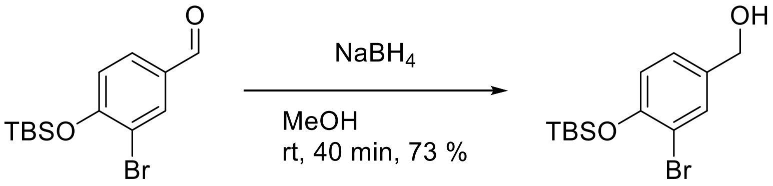

2 **3**

To a solution of 3-bromo-4-(tert-butyl-dimethylsilanyloxy)-hydroxybenzaldehyde **2** (3.39 g, 10.8 mmol) in dry MeOH (17 ml), $NaBH_4$ (203 mg, 5.38 mmol) was added portion wise. The reaction mixture was stirred at room temperature for 40 min. After completion, the reaction was quenched slowly with water. MeOH was removed under vacuum and extraction with DCM (3×15 ml) was carried out. The combined organic fractions were dried over $MgSO_4$ and concentrated under vacuum to yield a clear oil. The obtained crude product was purified by column chromatography (85:15 hexane:EtOAc) to afford (3-bromo-4-((tert-butyldimethylsilyl)oxy)phenyl)methanol **3** (2.50 g, 73%) as a clear oil.

^{1}H-NMR (400 MHz, CDCl3) δ 7.54 (d, J = 2.2 Hz, 1H), 7.16 (dd, J = 8.4, 2.2 Hz, 1H), 6.85 (d, J = 8.4 Hz, 1H), 4.59 (s, 2H), 1.04 (s, 9H), 0.24 (s, 6H).

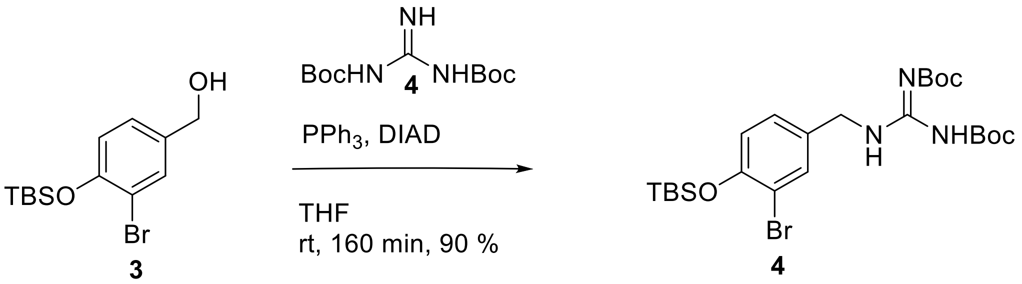

To a solution of (3-bromo-4-((tert-butyldimethylsilyl)oxy)phenyl)methanol **3** (993 mg, 3.15 mmol) in dry tetrahydrofuran (THF, 32 ml), 1,3-bis(tert-butoxy-carbonyl)-guanidine (1.23 g, 4.73 mmol), triphenylphosphine ($PPh_{3,}$ 23 g, 4.73 mmol) and Diisopropyl azodicarboxylate (DIAD, 931 µl, 4.73 mmol) were added. The reaction mixture was stirred for 100 min at room temperature. Reaction control via thin-layer chromatography (TLC) showed still starting material 3, therefore 0.5 eq. of each 1,3-bis(tert-butoxy-carbonyl)-guanidine (635 mg, 2.25 mmol), PPh_3 (613 mg, 2.34 mmol) and DIAD (445 µl, 2.26 mmol) were added. After another 60 min, the reaction was completed and concentrated under vacuum. The crude oil was purified by flash chromatography (95:5 hexane:EtOAc) to afford 1,3-bis(tert-butoxy-carbonyl)-(3-bromo-4-(tert-butyl-dimethyl-sylanoxy)-benzyl)-guanidine **4** (1.58 g, 90%) as a clear oil.

^{1}H-NMR (400 MHz, CDCl3) δ 9.54 (bs, 2H), 7.46 (d, J = 2.2 Hz, 1H), 7.24 – 7.16 (m, 1H), 6.81 (d, J = 8.4 Hz, 1H), 5.17 (s, 2H), 1.51 (s, 9H), 1.42 (s, 9H), 1.03 (s, 9H), 0.23 (s, 6H).

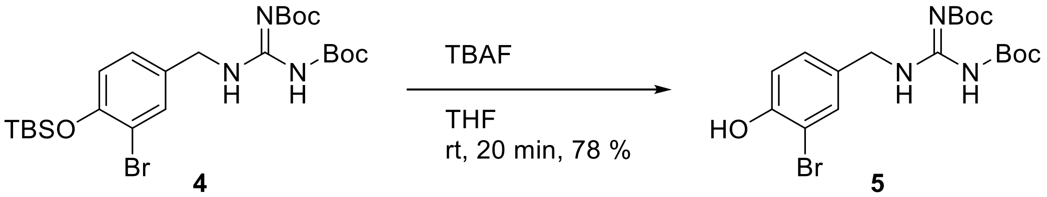

1,3-bis(tert-butoxy-carbonyl)-(3-bromo-4-(tert-butyl-dimethyl-sylanoxy)-benzyl)-guanidine **4** (2.05 g, 3.71 mmol) was dissolved in dry THF (48 ml). Tetrabutylammoniumfluoride (TBAF, 1 M in THF, 7.41 ml, 7.41 mmol) was added dropwise. The reaction mixture was stirred at room temperature for 20 min. Removing solvents under vacuum formed brownish viscous oil that was further purified using flash chromatography (25 → 50% EtOAc in hexane) resulting in 1,3-bis(tert-butoxy-carbonyl)-(3-bromo-4-hydroxy-benzyl)-guanidine **5** (1291 mg, 78%) as a white solid.

^{1}H-NMR (400 MHz, $CDCl_3$) δ 9.44 (bs, 2H), 7.41 (d, *J* = 2.0 Hz, 1H), 7.11 (dd, *J* = 8.4, 2.0 Hz, 1H), 6.89 (d, *J* = 8.4 Hz, 1H), 5.89 (bs, 1H), 5.06 (s, 2H), 1.50 (s, 9H), 1.40 (s, 9H).

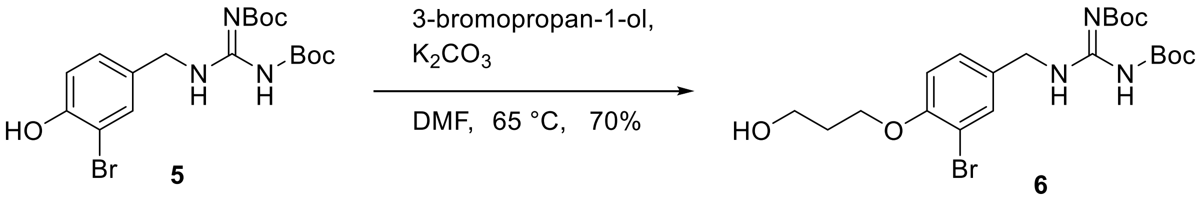

To a solution of 1,3-Bis(tert-butoxy-carbonyl)-(3-bromo-4-hydroxy-benzyl)-guanidine **5** (807 mg, 1.80 mmol) in dry DMF (20 ml), 3-bromopropan-1-ol (205 µl, 2.34 mmol) and K_2CO_3 (448 mg, 2.70 mmol) were added. The reaction mixture was heated to 60°C and stirred for 4.5 h. The reaction was quenched with the addition of H_2O (30 ml). Extraction with EtOAc (4×60 ml) was performed and the combined organic fractions were dried over $MgSO_4$ and concentrated under vacuum. The residue was purified by flash chromatography. After evaporation of the organic solvents of the product fractions, 1,3-bis(tert-butoxy-carbonyl)-(3-bromo-4-(3-hydroxo-propoxy)-benzyl)-guanidine **6** was obtained as a clear oil (634 mg, 70%).

^{1}H-NMR (400 MHz, $CDCl_3$) δ 9.48 (bs, 1H), 7.52 (d, *J* = 2.0 Hz, 1H), 7.31 (dd, *J* = 8.4 Hz, 2.0 Hz, 1H), 6.85 (d, *J* = 8.4 Hz, 1H), 5.13 (bs, 2H), 4.18 (t, *J* = 5.8 Hz, 2H), 3.91 (t, *J* = 5.8 Hz, 1H), 2.13 – 2.07 (p, *J* = 5.8 Hz, 2H), 1.51 (s, 9H), 1.43 (s, 9H).

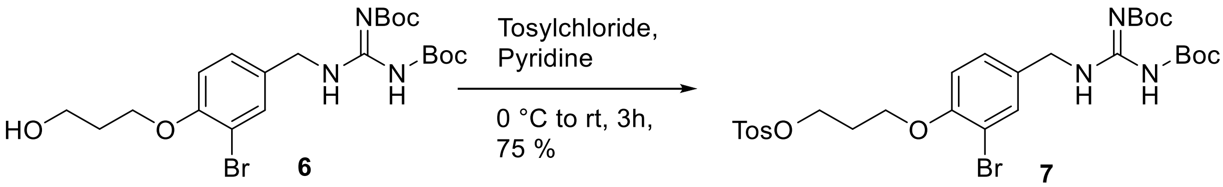

1,3-bis(tert-butoxy-carbonyl)-(3-bromo-4-(3-hydroxo-propoxy)-benzyl)-guanidine **6** (634 mg, 1.26 mmol) was dissolved in pyridine (2 ml) and cooled to 0°C. Tosylchloride (385 mg, 2.02 mmol) was added and the reaction mixture was allowed to warm up to room temperature. Reaction control was performed with TLC and no starting material was observed after 3 h. The reaction was stopped by the addition of H_2O and extraction with EtOAc (4× 60 ml) was performed. The combined organic fractions were combined and dried over $MgSO_4$ and concentrated under vacuum to afford 3-(4-((1,2-bis(tertbutoxycarbonyl)guanidino)methyl)-2-bromophenoxy)propyl-4-methylbenzenesulfonate **7** (623 mg, 75%) as a white solid.

^{1}H-NMR (500 MHz, $CDCl_3$) δ 9.42 (bs, 1H), 9.25 (bs, 1H), 7.72 (d, *J* = 8.2 Hz, 2H), 7.48 (d, *J* = 2.1 Hz, 1H), 7.19 (d, *J* = 8.2 Hz, 3H), 6.68 (d, *J* = 8.4 Hz, 1H), 5.06 (bs, 2H), 4.28 (t, *J* = 5.8 Hz, 2H), 3.95 (t, *J* = 5.8 Hz, 2H), 2.34 (s, 3H), 2.14 (p, *J* = 5.8 Hz, 2H), 1.49 (s, 9H), 1.41 (s, 9H).

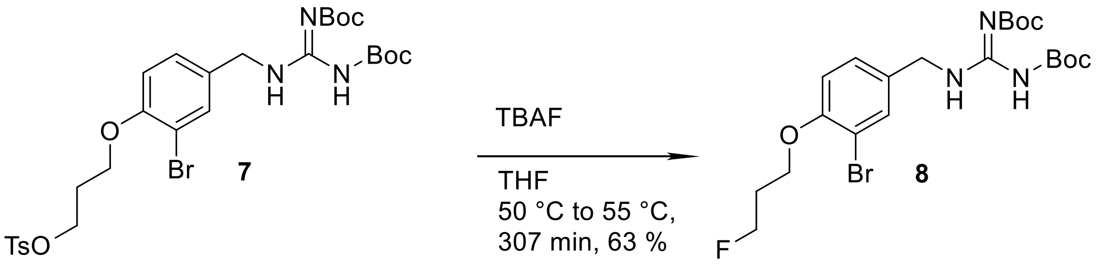

Intermediate **7** (102 mg, 0.16 mmol) was dissolved in dry THF (500 µl) and TBAF (1 M in THF, 310 µl, 0.31 mmol) was added. The reaction mixture was heated to 50°C and completion of the reaction could be observed after 5 h. Extraction with EtOAc (4×60 ml) was performed and the combined organic fractions were dried over $MgSO_4$ and concentrated *en vacuo* to yield a crude yellow oil. The crude product was purified by flash chromatography to afford 1-(3-bromo-4-(3-fluoropropoxy)benzyl)1,3-bis(tertbutoxycarbonyl)guanidine **8** (49 mg, 63%) as a yellowish oil.

^{1}H-NMR (400 MHz, $CDCl_3$) δ 9.63 (s, 1H), 7.59 (d, *J* = 2.2 Hz, 1H), 7.49 (d, 1H), 7.41 – 7.36 (m, 1H), 7.30 (dd, *J* = 8.4, 2.2 Hz, 1H), 6.86 (d, *J* = 8.4 Hz, 1H), 5.30 (bs, 2H), 4.76 (t, *J* = 5.8 Hz, 1H), 4.64 (t, 1H), 2.25 (p, *J* = 5.8 Hz, 1H), 2.20 – 2.15 (m, 1H), 1.52 (s, 9H), 1.48 (bs, 9H).

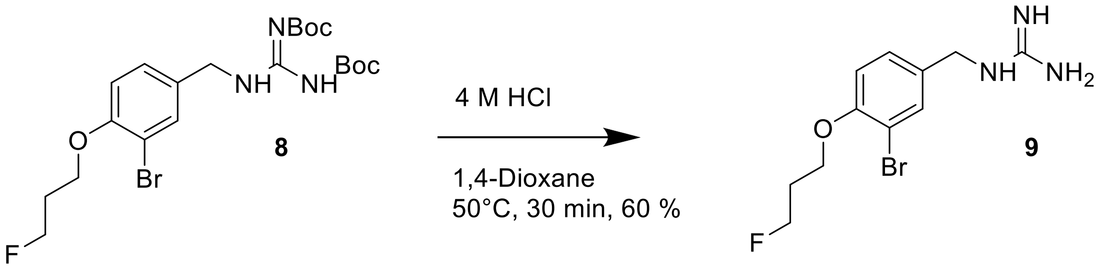

1-(3-bromo-4-(3-fluoropropoxy)benzyl)1,3-bis(tertbutoxycarbonyl)guanidine **8** (48 mg, 0.07 mmol) was dissolved in 1,4-dioxane (100 µl) and 4 M HCl (400 µl) was added. The reaction solution was heated to 50°C. After 30 min, the reaction was completed and the solution was neutralized to pH 4 using 4 M NaOH. Product **9** was purified by semi preparative HPLC using water (solvent A) and acetonitrile (solvent B) as solvent system and applying the following method: initial 100% A, 0% C, 0–20 min 80% A and 20% C, 20–25 min 80% A and 20% C, 25–26 min 100% A and 0% C, 26–30 min 100% A and 0% C. The flow was 4 ml/min and absorption measured at a wavelength of 254 nm. After lyophilization of the product fractions, reference compounds **9** was obtained as a white solid (22 mg, 59%).

^{1}H-NMR (400 MHz, MeOD) δ 7.54 (d, *J* = 2.2 Hz, 1H), 7.29 (dd, *J* = 8.4, 2.2 Hz, 1H), 7.07 (d, *J* = 8.4 Hz, 1H), 4.73 (t, *J* = 6.0 Hz, 1H), 4.67 (d, *J* = 47.2 Hz, 2H), 4.61 (t, 1H), 4.33 (s, 2H), 4.17 (t, *J* = 6.0 Hz, 2H), 2.22 (p, *J* = 6.0 Hz, 1H), 2.16 (p, *J* = 6.0 Hz, 1H).

^{13}C-NMR (101 MHz, MeOD) δ 156.39 (s), 133.35 (s), 131.40 (s), 128.99 (s), 114.64 (s), 113.23 (s), 82.30 (s), 80.68 (s), 66.03 (d, J = 6.6 Hz), 44.88 (s), 31.40 (d, J = 20.6 Hz).

HR-MS (ESI: M+H): calculated for $C_{11}H_{16}BrFN_3O$: 304.0455, m/z found was 304.0455.

Synthesis of [^{18}F]LMI1195

[^{18}F]fluoride ($^{18}F^-$) was produced via the $^{18}O(p,n)^{18}F$ nuclear reaction by bombardment of 98% enriched [^{18}O]water using a Cyclone 18/9 cyclotron (18-MeV; IBA, Belgium). Aqueous ^{18}F was trapped on a hydrophilic anion exchange cartridge (Waters SepPak Accell QMA cartridge carbonate) and eluted with a 2 ml acetonitrile/water (6:4) solution containing 10 mg kryptofix (K_{222}) and 1.2 mg K_2CO_3 into a reaction vessel. The solvents were evaporated at 90°C under reduced pressure with a gentle inflow of nitrogen gas. After addition of acetonitrile (MeCN, 1 ml), azeotropic drying was carried out. This procedure was repeated twice to afford dry K_{222}-K[^{18}F]F complex. A solution of precursor compound **7** (2 mg) in dry MeCN (0.5 ml) was added and the reaction mixture was stirred at 50°C for 30 min. The solvent was evaporated, and the reaction vessel was cooled down to 50°C, then HCl (1 ml, 4 M) was added for N-Boc deprotection and the reaction mixture was stirred for 10 min. After neutralization (0.8 ml NaOH, 4M) and dilution with 1.2 ml water, the crude product was purified by semi-preparative HPLC system by using an ACE column (250 x 10 mm). The collected product fraction was diluted with water (10 ml), trapped on a C18 light cartridge (Waters, preconditioned with 5 ml EtOH and 10 ml water), washed with water (5 ml) and eluted with acidified EtOH (0.5 ml, 1 µM HCl in EtOH) through a sterile filter (0.2 µm). The volume of EtOH was decreased under reduced pressure until ca. 0.1 ml and sodium phosphate buffer (0.15 M, pH 7.4, 2 ml) was added to give a final EtOH concentration of 5%. For quality control, an aliquot of the formulated solution was analysed using the analytical HPLC system. The identity of the ^{18}F-labeled product was confirmed by comparison with the HPLC retention time of its non-radioactive reference compound LMI1195 and by co-injection. The molar radioactivity of the product was calculated by comparison of UV peak intensity with a calibration curve of the non-radioactive reference compound.

Synthesis of [^{11}C]mHED

[^{11}C]CO_2 was produced in a Cyclone 18/19 cyclotron (18-MeV; IBA Belgium) via the $^{14}N(p,\alpha)^{11}C$ nuclear reaction and subsequently reduced by heterogeneous nickel catalysis as previously reported [1]. The resulting [^{11}C]methane was converted to [^{11}C]iodomethane by gas phase iodination and further reacted with silver triflate to afford the methylating agent [^{11}C]methyl triflate [2, 3]. The latter was bubbled into a solution containing 1 mg metaraminol in 0.4 ml of MeCN/H_2O (20:1) and the reaction mixture was stirred for 3 min at ambient temperature [4]. The mixture was diluted with 1.8 ml of aqueous NaH_2PO_4 (0.2 M) and purified by semi-

preparative HPLC (Luna C18 250 × 10.0 mm, 5 µm column) with the following conditions: 0.2 M NaH_2PO_4 in H_2O (solvent A), EtOH (solvent B); 0.0–1.9 min, 100% A; 2.0–2.1 min, 100–93% A; 2.2–14.9 min, 93% A; 15.0–15.1 min, 93–100% A; 15.2–25.0 min, 100% A. The flow rate was 5 ml/min and the UV signal was detected at 230 nm. The product was collected (retention time: 8.9 min) in a sterile vial and the pH of the final formulation was adjusted to 6.5 by the addition of sodium phosphate buffer (0.15 M, pH 7.4). Quality control was performed by HPLC using an ACE 3 C18 column (50 x 4.6 mm id) with the separation conditions: 0.1% TFA in H_2O (solvent A), MeCN (solvent B); 0.0–6.0 min, 2–5% B; 6.1–7.0 min, 5–2% B; 7.1–10.0 min, 2% B at a flow rate of 1 ml/min and UV signal detection at 280 nm (retention time: 5.6 min). Molar activities were calculated by comparison of UV intensity with a calibration curve of the corresponding non-radioactive standard.

Arteriovenous shunt and blood coincidence counting

For the arteriovenous shunt surgery, the mice were anesthetized with ~2.5% isoflurane in oxygen/air (50%/50%). Body temperature was maintained at 37°C with a heating pad. Polyethylene catheters (PE10 with an internal diameter of 0.28 mm; Smiths Medical, Adliswil, Switzerland) filled with heparinized (20 IU/ml) saline were inserted into the right femoral artery and vein with the help of a stereomicroscope and securely fastened with ligatures (6/0 suture thread, Fine Science Tools, Heidelberg, Germany). Additionally, catheters were held in place with tape. The animals were kept under anaesthesia for all subsequent procedures.

The arterial and venous catheters were connected and run through a coincidence counter (Twilite; Swisstrace GmbH, Zurich, Switzerland) positioned next to the scanner bed. The volume inside the blood counter was approximately 6 µl (10 cm of PE10). A constant flow of 120 µl/min was maintained by a peristaltic pump (Ismatec, Wertheim-Mondfeld, Germany). The tracer was injected into the arteriovenous shunt. The total volume of the tube system was approximately 60 µl. Blood counter data were recorded with the acquisition tool of the imaging software PMOD. The background counts of the blood counter were determined before tracer injection and were subtracted from the measured blood counts. The remaining counts were corrected for radioactivity decay with reference to the time point of tracer injection.

SUPPLEMENTARY TABLES AND FIGURES

Supplementary Table S1 Lower and upper limits of fit parameters

	K_1 (ml/min/cm^3)	k_2 (1/min)	k_3 (1/min) or K_3 (ml/min/cm^3)	k_4 (1/min)	v_b
**Lower limit**	0.006	0	0.006	0	0.1
**Upper limit**	2 (6 for initial evaluation)	2 (6 for initial evaluation)	2 (6 for initial evaluation)	1	0.9

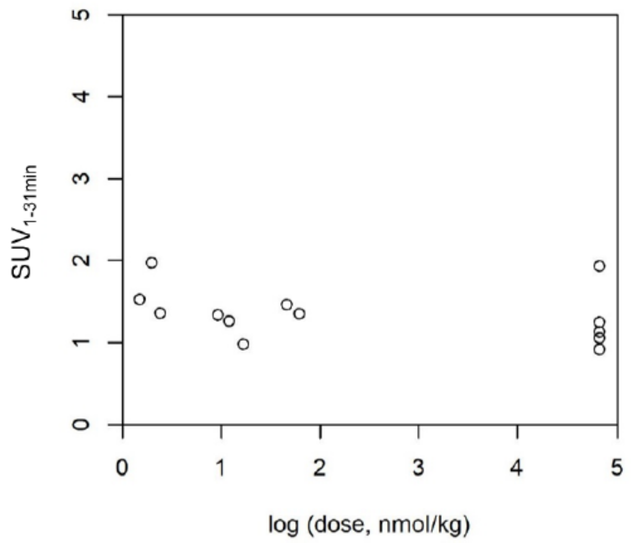

Supplementary Figure S1. SUV$_{1\text{-}31min}$ of the neck region was independent of NET inhibition by mHED, metaraminol or desipramine (combined dose on x axis).

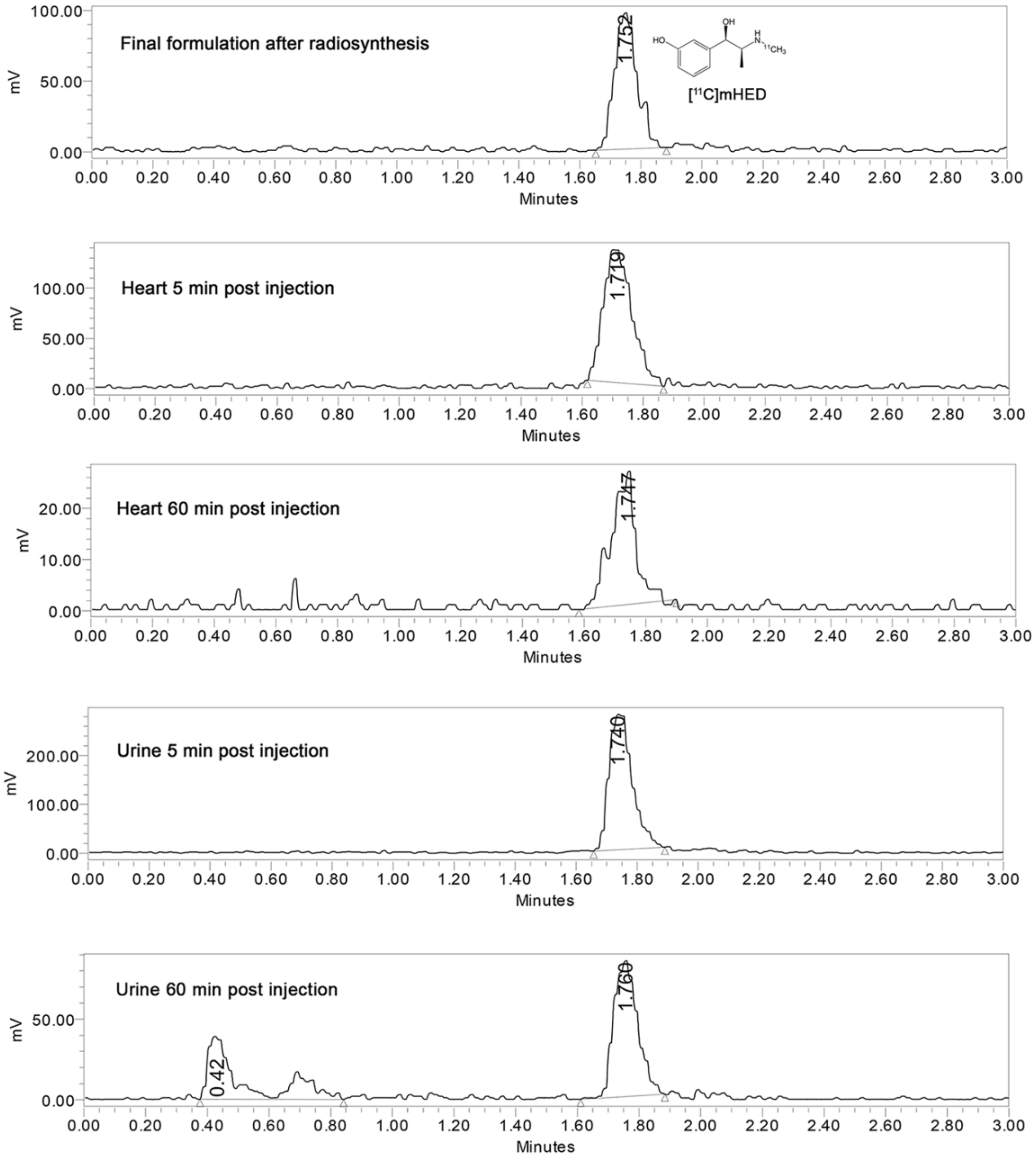

Supplementary Figure S2. Radio-UPLC profiles of heart and urine extracts at 5 and 60 min post injection of [^{11}C]mHED in FVN/B mouse. The top panel represents the formulated intact compound [^{11}C]mHED.

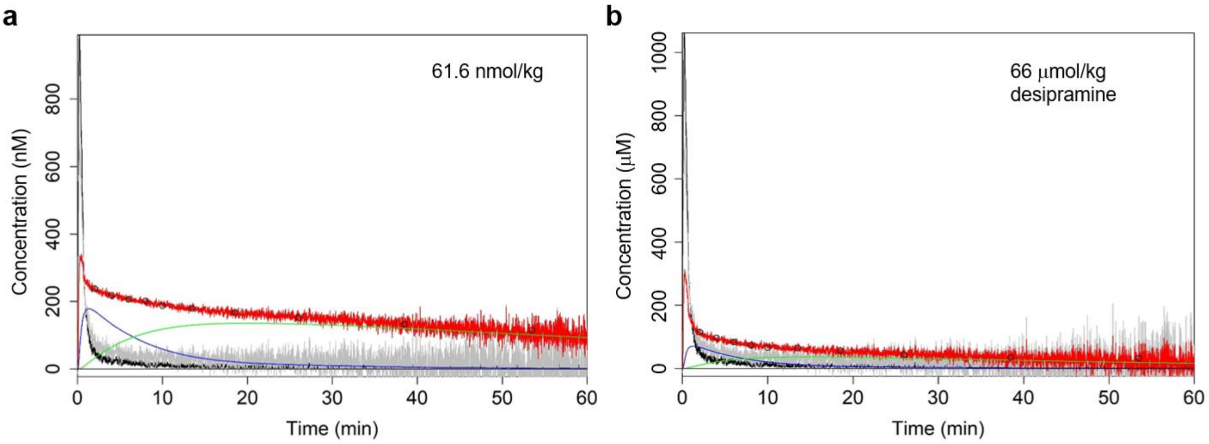

Supplementary Figure S3. TACs under partial or full NET saturation, fit based on a surrogate input function with the TCM2v. **a)** The injected combined mass of [^{11}C]mHED and metaraminol was 61.6 nmol/kg where partial NET saturation is expected. **b)** Scan with 66 µmol/kg desipramine i.p. pre-injection. Concentrations in nM (**a**) and µM (**b**) were calculated from the injected mass (mHED, metaraminol and desipramine) and measured radioactivity. Black circles, PET data (converted to concentrations); red lines, fitted TACs; black lines, surrogate input function; grey lines, corresponding whole blood radioactivity; blue and green lines, simulated concentrations in the individual tissue compartments of the TCM2v (see insert in **Figure 6d**).

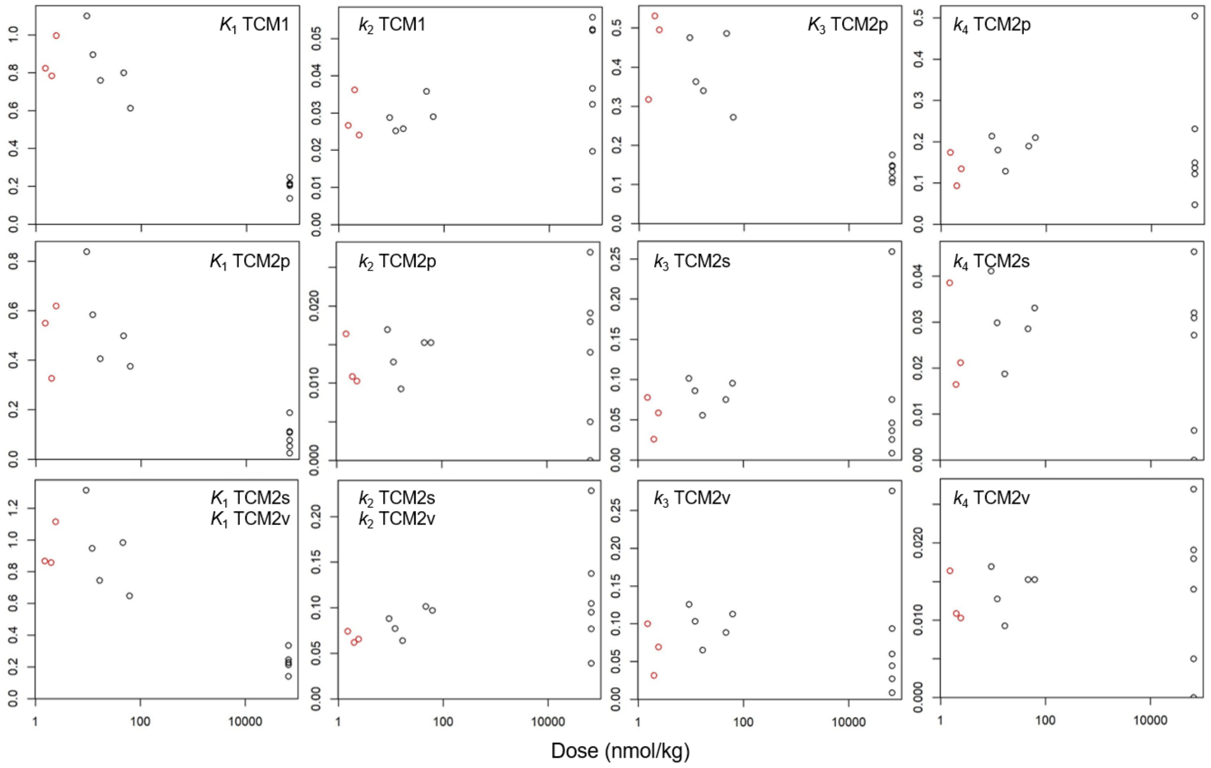

Supplementary Figure S4. Dose-dependence of fit parameters. Fit parameters of the four analyzed models (TCM1, TCM2p, TCM2s, and TCM2v) were compared to the total dose of combined mHED, metaraminol and desipramine. Symbols on the right dose scale (66 µmol/kg) are those with pre-administered desipramine (20 mg/kg as desipramine HCl). Red symbols, parameters from scans with an input function.

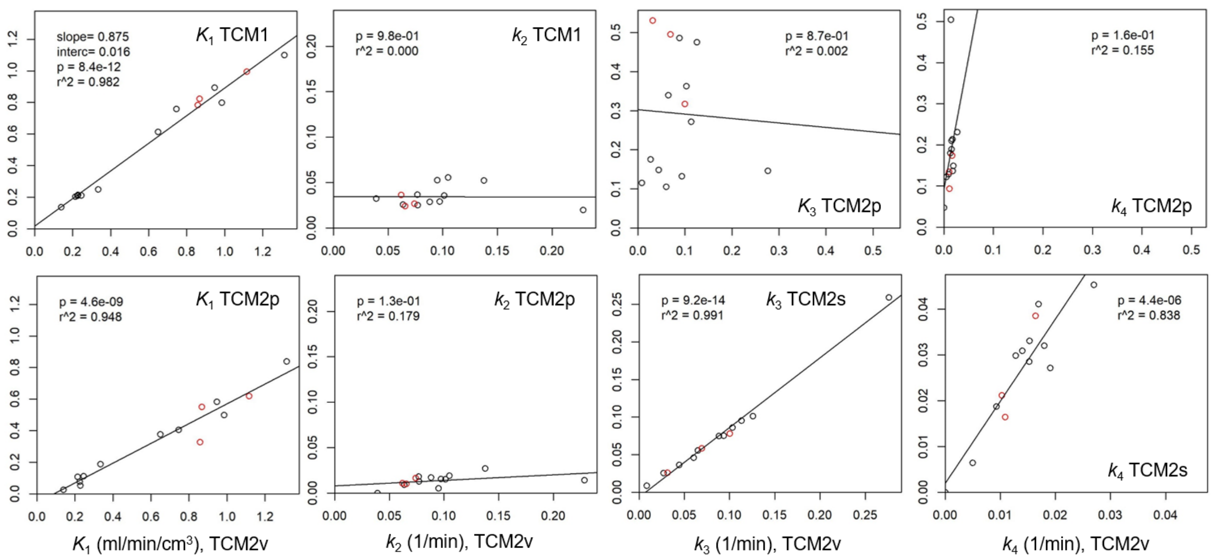

Supplementary Figure S5. Comparisons of clearance and mass transfer rate constants resulting from the fits to TCM1, TCM2p, TCM2s (y-axes), and TCM2v (x-axes). Doses of combined mHED, metaraminol and desipramine between 1.5 nmol/kg and 66 µmol/kg. Both K_1 and k_2 were equal for TCM2s and TCM2v. K_1 and K_3 in ml/min/cm^3; k_2, k_3, k_4 in 1/min. Top left plot as in **Figure 7b**. Red symbols, parameters from scans with an input function.

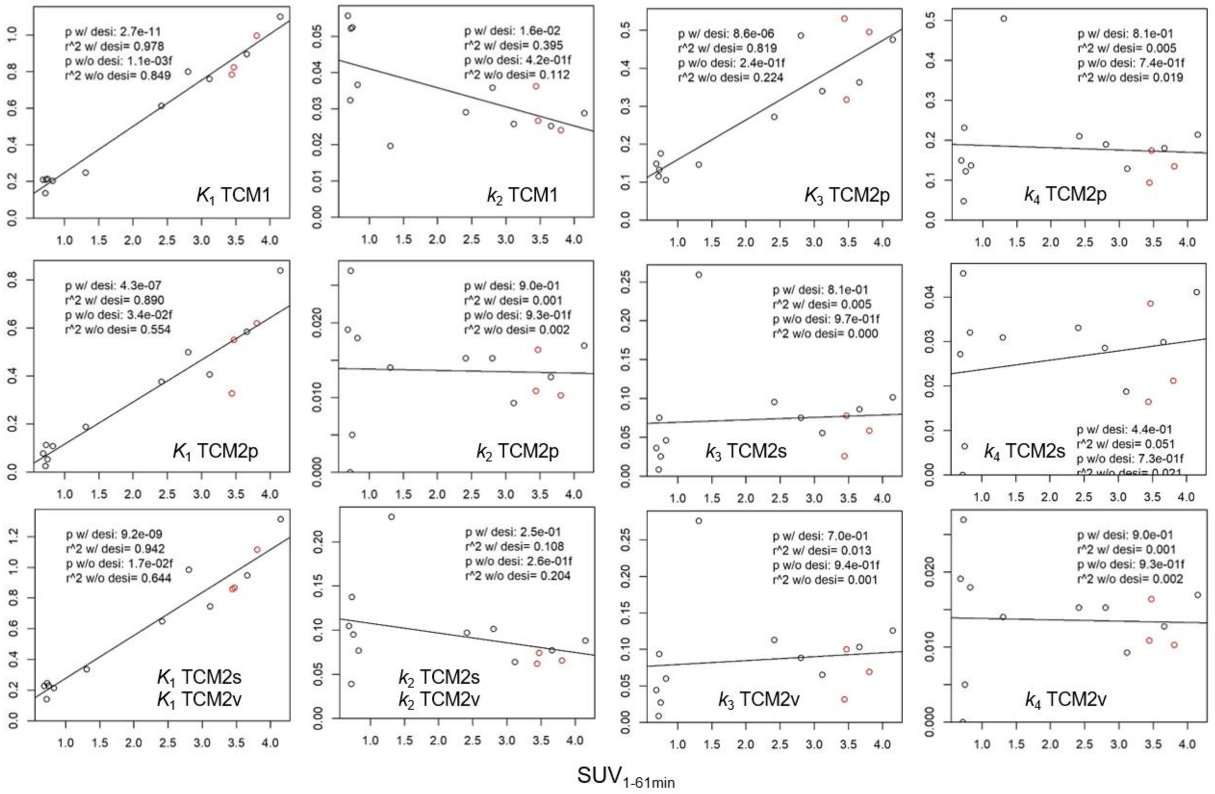

Supplementary Figure S6. Comparison of fit parameters K_1 to k_4 with model- and input-function independent $SUV_{1\text{-}61min}$. K_1 and K_3 in ml/min/cm^3; k_2, k_3, k_4 in 1/min. In the two-tissue compartment models, the correlation was significant for K_1, independent of whether desipramine scans were included or not (p<0.001 *vs* p=0.017; see **Figure 7c** for $SUV_{1\text{-}31min}$). It should be noted that 11 of the 14 scans were analyzed with a surrogate input function. Red symbols, parameters from scans with an input function.

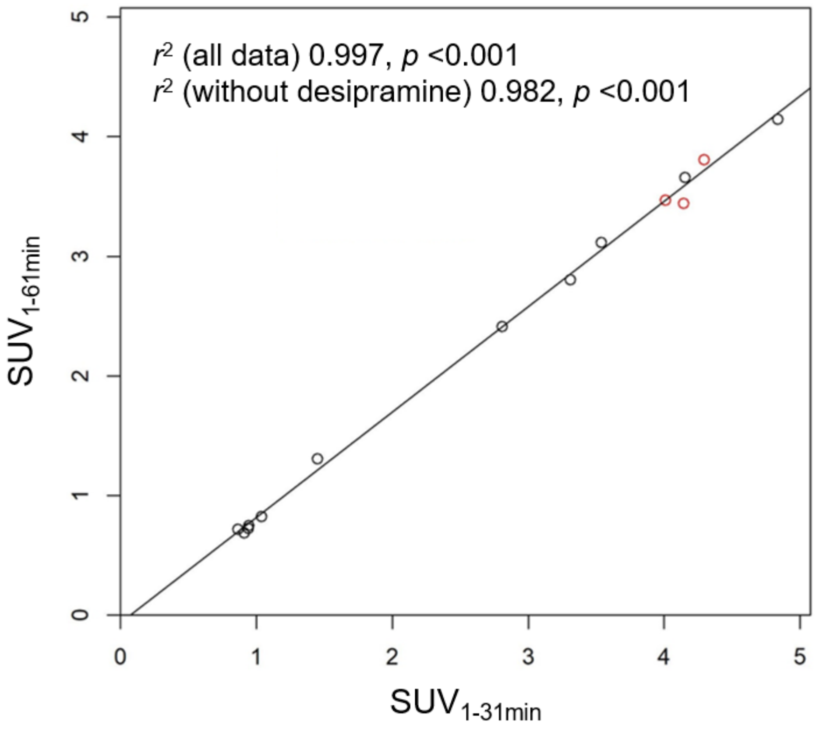

Supplementary Figure S7. Correlation between $SUV_{1\text{-}31min}$ and $SUV_{1\text{-}61min}$. r^2 and p as indicated. Line, linear regression. Red symbols, parameters from scans with an input function.

REFERENCES

1. Haider A, Spinelli F, Herde AM, Mu B, Keller C, Margelisch M et al. Evaluation of 4-oxo-quinoline-based CB2 PET radioligands in R6/2 chorea huntington mouse model and human ALS spinal cord tissue. European journal of medicinal chemistry. 2018;145:746-59. doi:10.1016/j.ejmech.2017.12.097.

2. Link JM, Krohn KA, Clark JC. Production of [11C]CH3I by single pass reaction of [11C]CH4 with I2. Nuclear medicine and biology. 1997;24(1):93-7.

3. Jewett DM. A simple synthesis of [11C]methyl triflate. International journal of radiation applications and instrumentation Part A, Applied radiation and isotopes. 1992;43(11):1383-5.

4. Rosenspire KC, Haka MS, Van Dort ME, Jewett DM, Gildersleeve DL, Schwaiger M et al. Synthesis and preliminary evaluation of carbon-11-meta-hydroxyephedrine: a false transmitter agent for heart neuronal imaging. Journal of nuclear medicine : official publication, Society of Nuclear Medicine. 1990;31(8):1328-34.
